# Deciphering the Role of π-Interactions in Polyelectrolyte Complexes Using Rationally Designed Peptides

**DOI:** 10.3390/polym13132074

**Published:** 2021-06-24

**Authors:** Sara Tabandeh, Cristina Elisabeth Lemus, Lorraine Leon

**Affiliations:** 1 Department of Materials Science and Engineering, University of Central Florida, Orlando, FL 32816, USA; sara.tabandeh@Knights.ucf.edu; 2 Department of Chemistry, University of Central Florida, Orlando, FL 32816, USA; clemus2@Knights.ucf.edu; 3 NanoScience Technology Center, University of Central Florida, Orlando, FL 32826, USA

**Keywords:** π-interactions, polyelectrolyte complexes, polypeptides, self-assembly, chirality, phase separation

## Abstract

Electrostatic interactions, and specifically π-interactions play a significant role in the liquid-liquid phase separation of proteins and formation of membraneless organelles/or biological condensates. Sequence patterning of peptides allows creating protein-like structures and controlling the chemistry and interactions of the mimetic molecules. A library of oppositely charged polypeptides was designed and synthesized to investigate the role of π-interactions on phase separation and secondary structures of polyelectrolyte complexes. Phenylalanine was chosen as the π-containing residue and was used together with lysine or glutamic acid in the design of positively or negatively charged sequences. The effect of charge density and also the substitution of fluorine on the phenylalanine ring, known to disrupt π-interactions, were investigated. Characterization analysis using MALDI-TOF mass spectroscopy, H NMR, and circular dichroism (CD) confirmed the molecular structure and chiral pattern of peptide sequences. Despite an alternating sequence of chirality previously shown to promote liquid-liquid phase separation, complexes appeared as solid precipitates, suggesting strong interactions between the sequence pairs. The secondary structures of sequence pairs showed the formation of hydrogen-bonded structures with a β-sheet signal in FTIR spectroscopy. The presence of fluorine decreased hydrogen bonding due to its inhibitory effect on π-interactions. π-interactions resulted in enhanced stability of complexes against salt, and higher critical salt concentrations for complexes with more π-containing amino acids. Furthermore, UV-vis spectroscopy showed that sequences containing π-interactions and increased charge density encapsulated a small charged molecule with π-bonds with high efficiency. These findings highlight the interplay between ionic, hydrophobic, hydrogen bonding, and π-interactions in polyelectrolyte complex formation and enhance our understanding of phase separation phenomena in protein-like structures.

## 1. Introduction

The association of oppositely charged polymers in aqueous solutions results in phase-separated liquid droplets called complex coacervates, or amorphous solid precipitates [1,2,3]. Entropy gain from the release of adsorbed counterions and dehydration is believed to be the main driving force for complexation [4,5,6,7,8,9]. However, short-range forces such as hydrogen bonding and hydrophobic interactions can also be involved in the complex formation process [10,11,12,13,14,15]. Hydrogen bonding between the sequence pairs results in strong interactions and the formation of a compact structure. However, introducing steric effects via sequence patterning can disrupt hydrogen bonding and the physical state of the complexes. For example, heterochirality in ionic peptide pairing promotes liquid coacervation [10]. Hydrophobicity also affects the polyelectrolyte complexation process. We previously showed that hydrophobicity can control phase behavior and properties of polyelectrolyte complexes, resulting in improved stability against salt and temperature [11].

Cation-π and π-π interactions are among the non-covalent interactions that play important roles in biology and the design of self-assembled materials [16,17]. A π-π interaction is the attractive force between aromatic rings due to the presence of π-electron clouds [18], while a cation-π interaction is the attraction force between the positive charge of a cation and the negative face of an aromatic ring [19]. However, some geometric configurations are preferred in driving the π-interactions of proteins. Hunter et al. studied structural orientations of π-π interactions in proteins using phenylalanine residues in their analysis [20]. They reported a dominance of electrostatically favorable geometries such as edge-to-face, and concluded that face-to-face orientations are less favorable in the π-stacking of aromatic rings. Similar to polyelectrolyte complexes, the release of ordered water molecules as the result of intermolecular interactions causes π-interactions to be entropically driven [18].

π-interactions play an important role in biological liquid–liquid phase separation [21,22,23,24]. Biomolecular condensates such as nucleoli, P bodies, and stress granules are formed through liquid-liquid phase separation and lack a surrounding membrane [25]. These membraneless condensates are enriched in proteins and nucleic acids, performing versatile functions such as signaling and protection of cellular components [26]. The lack of membrane allows rapid response to the environmental changes, and their selective compartmentalization and entry of biomolecules to the condensates distinguish them from membrane-bound organelles such as mitochondria, nuclei, and Golgi apparatuses [27,28,29]. The importance of cation-π interactions on the stability of membraneless organelles has been shown in a study by Nott et al. [21]. They showed that electrostatic attraction between oppositely charged residues and cation-π interactions between repeated sequences of phenylalanine-glycine (FG) and arginine-glycine (RG) govern the phase separation of Ddx4 proteins. They also realized that FG sequences are spatially close to positively charged dipeptides (RG), and substitution of aromatic (phenylalanine, F) with non-aromatic residues (alanine, A) disrupts phase separation. The supportive effect of cation-π interactions in phase separation of proteins has also been shown by cation-π pairing of arginine with tyrosine residues of the RNA binding protein FUS [30]. In addition to liquid droplets, self-assembly in biological systems can lead to aggregation. Well-known examples of these aggregate formations are amyloid fibrils that cause diseases such as Alzheimer’s, type II diabetes, and prion diseases [31]. Aromatic residues have been found in amyloid-related polypeptides, supporting the role of π-interactions in amyloid fibril formation.

Das et al. evaluated physical forces that affect liquid–liquid phase separation of proteins using coarse-grained residue-based protein chain models [32]. They pointed out that π–π interactions have a supportive effect on the hydrophobic, electrostatic, and cation–π interactions in the phase separation process. For instance, the guanidinium group of arginine packs with aromatic rings and provides stronger arginine–π over lysine–π contacts, resulting in a more favorable interaction with arginine over lysine. Otherwise, considering only their charge, arginine and lysine would have similar average contact energies [33].

Furthermore, π-interactions can tune the self-assembly process of materials. In a study by Ghosh et al. [34], pyrene conjugation to phenylalanine-phenylalanine (FF) dipeptides resulted in β-sheet-rich structures due to the synergistic effect of π-interactions and intermolecular hydrogen bonding. On the contrary, another chromophore with fewer aromatic rings, naphthalene, did not show effective π-stacking upon conjugation to FF and showed similar behavior to the control molecule without chromophore. The stabilization effect of π-interactions on self-assembled peptides was also shown by Wychowaniec et al., where they reported that using phenyl-glycine residues in ionic tetra-peptides helps stabilize β-sheet domains through the formation of intermolecular π-stacking and a compact structure [35]. π-interactions can also drive the self-assembly of like-charged polyelectrolytes such as mussel foot protein-1 and poly(2-(trimethylamino)ethyl methacrylate), both positively charged, via cation-π bonding [36]. The trimethylammonium group interacts with the tyrosine of mussel foot protein-1 and establishes cation-π bonding. Additionally, π-interactions have been used in biomaterials design such as self-assembled hydrogels used for 3-D cell culture applications. Upon self-assembly, fluorenylmethoxycarbonyl (FMOC)-aromatic dipeptides have shown stability at physiological conditions and cell proliferation support due to their π-interactions [37].

Given the significance of π-interactions in biological systems, it is necessary to understand the effect of cooperative interactions that control the phase separation process. In a recent study, Kapelner et al. reviewed molecular features, including electrostatic, hydrophobic, and aromatic interactions, that govern the phase separation process of polyelectrolytes and proteins in synthetic and cellular systems [38]. We previously evaluated the effect of hydrophobicity on the phase separation behavior of polyelectrolyte complexes [11]. We used a rational design of hydrophobically-patterned ionic polypeptides and showed that enhanced hydrophobicity results in more stable complexes with higher encapsulation efficiency for a model hydrophobic molecule.

Current work focuses on polypeptide complex formation where other interactions such as π-π and cation-π are also involved. We apply a sequence patterning of charged and non-charged residues to synthesize a library of six polypeptides. Phenylalanine is used as the non-charged residue due to its hydrophobicity and aromatic ring in the side chain. We also study the effect of increased charge density by modifying the sequence to include more charged residues than aromatic. We then change the non-charged site by placing a phenylalanine derivative with a fluorine substituent on the aromatic ring which has been shown to disrupt π-interactions [39]. Then, we evaluate how π-interactions, together with charge interactions, hydrogen bonding, and hydrophobic interactions, influence polypeptide complex formation. Furthermore, we compare the compartmentalization behavior of complexes using a model dye, methylene blue, that contains delocalized electrons, and is not highly encapsulated while relying just on electrostatic interactions [40].

## 2. Materials and Methods

### 2.1. Materials

All polypeptide sequences were synthesized using solid-phase peptide synthesis (SPPS) on a peptide synthesizer (PS3, Gyros Protein Technologies Inc., Tuscan, AZ, USA). All amino acids for the synthesis were fluorenylmethyloxycarbonyl (FMOC)- protected and were purchased from Chem-Impex International Inc. (Wood Dale, IL, USA). 2-(1H-benzotriazole-1-yl)-1,1,3,3-tetramethyluronium hexafluorophosphate (HBTU) (Oakwood Chemical, SC, USA) was used as an activator. Solid support for the synthesis process was an FMOC-Rink Amide resin purchased from Novabiochem (MA, USA). Solvents for the synthesis process were dimethylformamide (Fisher Scientist, Fair Lawn, NJ, USA), N-methylmorpholine, and piperidine (Sigma-Aldrich, St. Louis, MO, USA). A mixture of Trifluoroacetic acid (Fisher Scientist, Fair Lawn, NJ, USA), Triisopropylsilane (TIS) (Acros, NJ, USA), and water (95:2.5:2.5 % volumetric ratio, respectively) was used to cleave the peptide from the resin and to remove side-chain protecting groups. Precipitation of peptides was performed in cold diethyl ether as the last step to have the final peptide product. All peptides were stored at −20 °C.

Three polycation sequences, poly(lysine-glycine) (p(kG)), poly(lysine-alanine) (p(kA)), and poly(lysine-leucine) (pkL), with a degree of polymerization of 30 and alternating chiral pattern, were previously synthesized using the same synthesis process. The characterization of these sequences was previously published [11].

Sodium chloride (NaCl) and calcium chloride dihydrate (CaCl_2_·2H_2_O) were purchased from Fisher Scientific (Fair Lawn, NJ, USA). Methylene blue was purchased from Acros Organics (Fair Lawn, NJ, USA).

### 2.2. Polyelectrolyte Complex Preparation

Complex samples were prepared at a 1:1 molar ratio (monomer charge basis) of polyanions: polycations. The polyanion was added first to the water or water-salt solution, followed by the polycation and then vortexing for better mixing. The total concentration of polymers (charge basis) in the final solution was 5 mM, unless stated otherwise. Stock solutions of the polyanion and the polycation had a concentration of 10 mM, monomer charge basis, and were kept at 4 °C. The pH of the solutions was adjusted to 7 using sodium hydroxide or hydrochloric acid solutions, assuming that polyanions and polycations are fully charged at this pH as it is two units away from the pKa value of both polylysine and polyglutamic acid (pKa values of polyglutamic acid and polylysine are around 4.3 and 10, respectively) [41,42].

### 2.3. Nuclear Magnetic Resonance (NMR) Spectroscopy

H NMR spectroscopy of synthesized sequences was carried out on a Bruker Avance III 400 HD MHz spectrometer. Samples were prepared in D_2_O with a concentration of 10 mg/mL.

### 2.4. Matrix Assisted Laser Desorption/Ionization Time of Flight (MALDI-TOF) Mass Spectrometry

Polypeptide molecular weight was determined using a Bruker Microflex KRF MALDI-TOF mass spectrometer (Fremont, CA, USA). Samples were loaded on a 96-spot target plate (MSP 96 target ground steel) using α-cyano-4-hydroxycinnamic acid as the matrix.

### 2.5. Circular Dichroism (CD)

The secondary structure and chirality pattern of the polypeptides were investigated using a CD spectrophotometer (Olis DSM 20, Bogart, GA, USA). Samples were prepared in aqueous solutions with a concentration of 0.08 or 1 mg/mL. All spectra were collected from 190 nm to 250 nm at room temperature in a cuvette of 0.1 cm pathlength. Spectra were the average of 5 scans at 1 nm intervals. All data were collected in millidegrees. Data were then converted and reported as mean residue ellipticity (MRE, mdeg.cm^2^/dmol) to normalize against concentration.

### 2.6. Attenuated Total Reflection-Fourier Transform (ATR-FTIR) Spectroscopy

ATR-FTIR measurements of individual polypeptides, as well as their complexes, were performed on a Shimadzu IRSpirit FTIR spectrometer (Shimadzu, MD, USA). All FTIR spectra (600 cm^−1^ to 4000 cm^−1^) were recorded at a spectral resolution of 4 cm^−1^ with an average number of scans of 120 at room temperature. The background scan of the empty crystal in the air was taken before each sample run and subtracted by the software. Samples were prepared in D_2_O with a final concentration of 100 mM (monomer charge basis). D_2_O spectrum was subtracted manually.

### 2.7. Absorbance Measurements

Absorbance measurement of polypeptide complexes was conducted on a Cytation5 imaging reader (Biotek Inc., Winooski, VT, USA), equipped with an ultraviolet spectrophotometer. For turbidity measurements, absorbance was recorded at 500 nm wavelength. Polypeptides do not absorb light at this wavelength. The relative turbidity is defined as −ln(I/I_0_), where I_0_ is the incident light intensity, and I is the light intensity that passes through the sample volume. The turbidity was reported in absorption units (a.u.). Three measurements were performed for each sample and averaged, where the error bars on turbidity plots represent the calculated standard deviation of the data.

Ultraviolet-visible (UV-vis) spectra were collected using the same plate reader. Absorbance spectra of the dye and supernatant solutions were recorded at room temperature using a wavelength range of 300 to 800 nm.

A dark 96-well plate with transparent bottom was used for absorbance measurements (Costar, Corning Inc. Kennebunk, ME, USA). An aliquot of 120–150 µL of each sample was dispensed into the 96-well plate. Samples were prepared at a total concentration of 5 mM with respect to the monomer charge and were used immediately after the final step of mixing (as per Section 2.2).

### 2.8. Optical Microscopy

The physical state of polyelectrolyte complexes was examined on a Cytation5 imaging reader (Biotek Inc., Winooski, VT, USA), using a 20× objective in bright-field mode. Samples were prepared as described in Section 2.2, placed in a 96-well plate, and imaged after 15 min. For fluorescence imaging of samples with thioflavin, a DAPI filter with an excitation wavelength of 377 nm and an emission of 447 nm was used.

## 3. Results and Discussion

### 3.1. Sequence Patterning of Charged Phenylalanine Peptides

**Figure 1:**
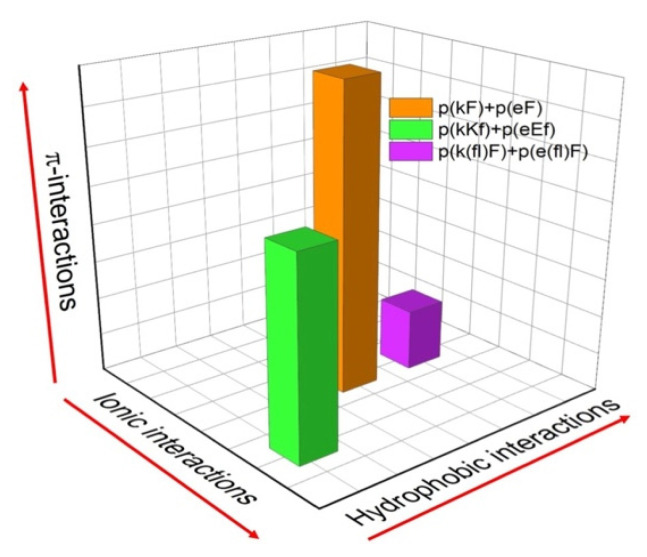
Relative comparison of the different molecular interactions present in the designed peptide sequence pairs. The magnitudes of each interaction (hydrophobic, ionic, and π-interactions) are approximate for the sake of comparison.

We have designed three sequence pairs consisting of a polyanion and polycation with phenylalanine and its derivative, 4-fluoro-phenylalanine (Table 1). Each sequence pair has 30 amino acid residues and is synthesized by solid phase peptide synthesis (SPPS). The first sequence pair has an alternating pattern of D-lysine or D-glutamic acid and L-phenylalanine ((kF) and (eF), respectively). The second sequence pair has a higher charge density by placing two lysines or two glutamic acids in a structural unit. The last sequence pair has a similar structural design as the first sequence (one charged and one non-charged residue), but we have substituted phenylalanine with 4-fluoro-phenylalanine ((fl)F). All molecules are summarized in Table 1. A relative comparison of the different molecular interactions present (ionic, hydrophobic, π) in the designed sequence pairs is shown in Figure 1. The reason for the alternating use of D and L-chiral amino acids in our designs is because alternating chiral patterns can inhibit hydrogen bonding and thus promote liquid coacervate phases using peptide-based polyelectrolytes [10,43,44]. The molecular weight of synthesized polypeptides was determined by MALDI-TOF mass spectrometry. The measured mass to charge ratio of samples verifies the expected molecular weight and accuracy of the synthesis process (Supplementary Figure S1 and Supplementary Table S1). A small variation in the measured molecular weight versus the expected molecular weight can be due to the presence of counterions. Here, the difference between measured molecular weights and theoretical values is between 24 to 29 Daltons. We attribute this difference to the mass of sodium ions present in the matrix-sample mixtures [45]. H NMR was used to assess the structural composition and correct ratio of amino acids in each peptide sequence. The molecular structures and the signals assigned to hydrogens within molecular structures are shown in Supplementary Figure S2, confirming the correct proportion of amino acids.

### 3.2. Circular Dichroism (CD) of Phenylalanine Peptide Sequences

**Figure 2:**
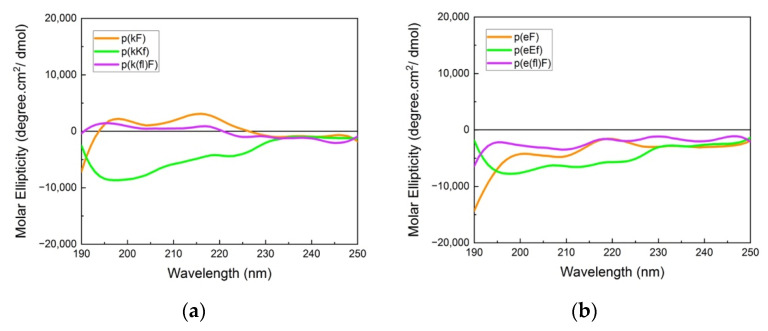
Circular dichroism (CD) spectra of (**a**) polycation and (**b**) polyanion sequences. Signal cancellation from opposite chirality results in almost a flat signal; otherwise, due to the like-charged groups on each patterned sequence, a random coil signal (usually with a minimum (or maximum for D-chirality) at 195 nm) can be observed.

The far-UV CD spectra (190–250 nm) provide information on the chiral pattern and secondary structure of polypeptide sequences. CD spectra of highly charged polypeptides usually show a random coil signal due to the repulsion of like-charged groups with a characteristic minimum around 195 nm. However, the designed peptide sequences in our work have an alternating pattern of D and L-chirality, which leads to canceling of the difference between left and right circularly polarized light leading to a flat CD absorbance [44], hence, making the secondary structure interpretation challenging. Absorbance spectra of polycations and polyanions are shown in Figure 2. Polypeptides with (kX) and (eX) patterns (X being phenylalanine or 4-fluoro-phenylalanine) indicate a nearly flat absorbance due to signal cancellation arising from the alternating chirality of the sequence. Fluorinated phenylalanine sequences, p(k(fl)F) and p(e(fl)F), demonstrated almost the same spectra compared to the non-fluorinated counterparts, p(kF) and p(eF). This result suggests that fluorine substitution on the phenylalanine ring of (kF) and (eF) might not affect their secondary structure. Budisa et al. observed similar behavior in their work on Candida Antarctica lipase B. The secondary structure profile of Candida Antarctica lipase B did not change by replacing phenylalanine residues with 4-fluoro-phenylalanine [46]. p(kKf) and p(eEf) sequences show a similar flat absorbance profile as other sequences and a minimum near 195 nm.

### 3.3. Secondary Structure Analysis of Polypeptides and Sequence Pairs

**Figure 3:**
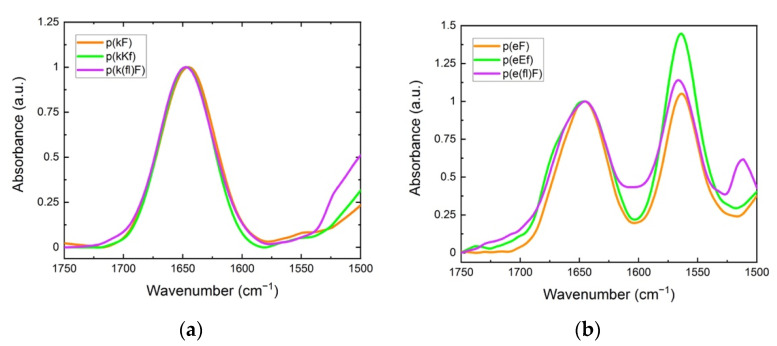
FTIR spectra: (**a**) polycation; (**b**) polyanion sequences. The peak at around 1645 cm^−1^ is characteristic of the unordered (random coil) conformation. The signal at 1564 cm^−1^ is due to the sidechain stretching of the carbonyl group of glutamic acid. The signal for the carbon-carbon stretching vibration of the fluoro-phenylalanine aromatic ring is observed at 1510 cm^−1^.

FTIR spectroscopy was used to elucidate the cooperative effect of π-interactions, electrostatic, and hydrophobic forces on the structural conformation of polypeptides and their sequence pairs. The carbonyl stretching vibration of the peptide’s backbone gives rise to signals in the amide I region (1600 to 1700 cm^−1^) using FTIR spectroscopy, providing information on the secondary structure [47]. In the amide I region, secondary structure sensitivity arises from hydrogen bonding of the backbone carbonyl groups and the transition dipole coupling of neighboring amide groups [48,49]. The absorption bands in this region are characteristic of different structural conformations of peptides such as random coil, α-helix, β-sheet, and β-turn [50]. Individual sequences, as shown in Figure 3, show a peak at 1645 cm^−1^, indicating an unordered structure [51]. For polyanion sequences, there is another peak at 1564 cm^−1^ attributed to the carbonyl stretching of the glutamic acid sidechain. The signal peak at around 1510 cm^−1^ is associated with the carbon-carbon stretching vibration in the aromatic ring of fluoro-phenylalanine sequences [52]. Trifluoroacetic acid (TFA) binds to polycations after the cleavage process and remains as their counterion. TFA shows a strong signal at 1673 cm^−1^ which can interfere with the β-sheet absorption band at around 1680 cm^−1^ [53,54]. Therefore, we replaced the TFA counterion of the polycations with a chloride ion by repeating a dissolution-lyophilization procedure, 3–4 times, in a dilute hydrochloric acid (HCl) solution (5 mM).

**Figure 4:**
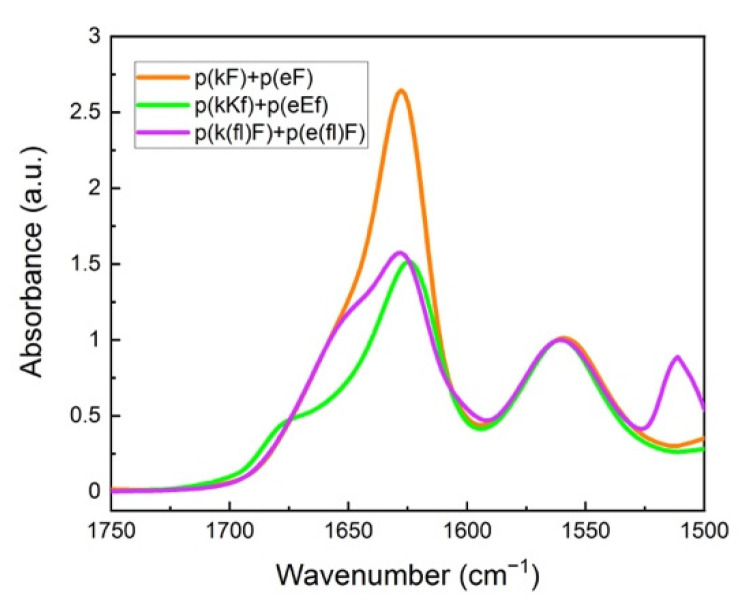
FTIR spectra of sequence pairs: p(kF) + p(eF), p(kKf) + p(eEf), and p(k(fl)F) + p(e(fl)F). The peak at around 1625 cm^−1^ is characteristic of β-sheet formation, overlapping with the random coil conformation signal at around 1645 cm^−1^. Glutamic acid sidechain carbonyl stretching is observed at 1564 cm^−1^. The signal at 1510 cm^−1^ and a low-intensity shoulder at 1603 cm^−1^ are associated with the carbon-carbon stretching vibration of the fluoro-phenylalanine aromatic ring.

Figure 4 shows FTIR spectra of the polypeptide pairs. A peak at around 1625 cm^−1^ is observed for all systems and is characteristic of β-sheet formation [47]. We performed deconvolution analysis of the amide I spectral region of sequence pairs to obtain more detailed information on the overlapped peaks (Supplementary Figure S3). Curve-fitting analysis was performed using Origin software. Band assignments and attributed secondary structure are summarized in Table 2. An amide I peak in the range of 1615–1640 cm^−1^ corresponds to β-sheet formation [54,55]. Deconvolution analysis of the p(kF) + p(eF) spectrum presents the highest amount of β-sheet conformation than other sequence pairs, followed by the higher charged-density pair, p(kKf) + p(eEf), and then the fluorinated-phenylalanine sequence pair, p(k(fl)F) + p(e(fl)F).

These patterned sequences contain both charged and hydrophobic aromatic residues, providing an opportunity for charge-charge, cation-π, π-π, and hydrophobic interactions. The interplay among these interactions can influence the stability of polyelectrolyte complexes. p(kF) + p(eF) has all possible interactions, i.e., ionic, cation-π, π-π, and hydrophobic pairing, leading to increased hydrogen bonding. p(kKf) + p(eEf), on the other hand, with higher charge density, is less hydrophobic and has less aromatic content (33.3% vs. 50%) compared to (kF) + (eF) system. Furthermore, the presence of two identical amino acids with opposite chirality in the structural unit of p(kKf) + p(eEf) can disrupt hydrogen bonding to a greater extent than p(kF) + p(eF). We previously showed that such sequence pattering disrupts hydrogen bonding and causes liquid complex formation, by comparing p(kA) + p(eA) with p(kKa) + p(eEa) [11]. Here, the interplay between ionic, hydrophobic, and π-interactions can lead to a synergetic effect resulting in the stabilization and aggregation of these complexes. Increasing hydrophobic and π-interactions has been reported to cause stronger bonds and solid formation in the RNA-binding protein FUS when Murthy et al. replaced all tyrosine residues with phenylalanine [56]. p(k(fl)F) + p (e(fl)F) has a fluorine substituent on the aromatic ring of the phenylalanine residues which has an inhibitory effect on the cation-π interactions [39]. The high electronegativity of fluorine withdraws electron density from the phenylalanine ring and hence, weakens the cation–π attraction. However, other interactions such as electrostatic forces and hydrophobic effects contribute to the stabilization and aggregation of p(k(fl)F) + p(e(fl)F).

Stabilization of phase-separated intrinsically disordered proteins by π-interactions has been reported previously. In a study on histidine-rich squid beak proteins (HBPs), Gabryelczyk et al. showed that condensation of HBPs initiates primarily by deprotonation of histidine and hydrogen bonding with tyrosine residues, and their stabilization depends on following π-stacking of tyrosine residues [57].

**Figure 5:**
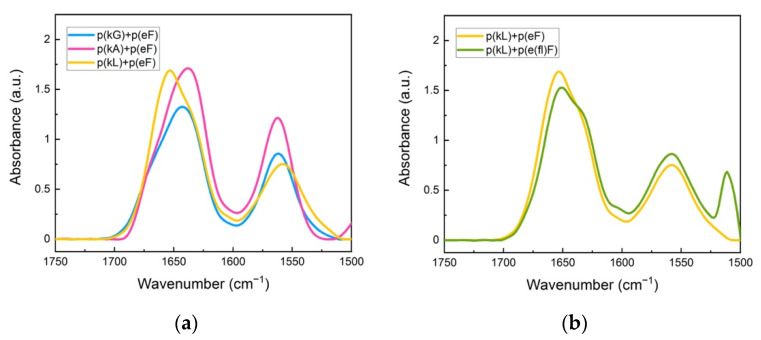
FTIR spectra of sequence pairs: (**a**) p(kG), p(kA), and p(L) with p(eF); (**b**) p(kL) sequence paired with p(eF), and p(e(fl)F). All sequence pairs show the characteristic random coil signal at around 1645 cm^−1^. The signal at 1564 cm^−1^ is due to the sidechain stretching of the carbonyl group of glutamic acid. The peak at 1510 cm^−1^ and also a low intensity signal at around 1603 cm^−1^ are indicative of carbon-carbon stretching vibration in the aromatic ring of fluoro-phenylalanine.

We further explored how decreased π-interactions between the polypeptide pairs can affect the secondary structure of complexes. We selected three ionic sequences with varying hydrophobic content, already made in-house [11], as a polypeptide pair with the p(eF). Figure 5 shows FTIR spectra of p(kG), p(kA), and p(kL), with increasing hydrophobic content, respectively, pairing with the p(eF) sequence. Deconvolution of the FTIR spectra is shown in Supplementary Figure S4.

The secondary structure analysis of deconvoluted spectra is shown in Table 3. These sequence pairs show less overall β-sheet formation than previous complexes, where both sequences have aromatic residues. We believe that the lack of π-π interactions between the polypeptide pairs results in weaker interactions and less β-sheet formation even though the cation-π interactions are still present. p(kL) + p(eF) is the only pair with an assignment band at around 1625 cm^−1^ compared to the sequence pairs of p(kG) + p(eF), and p(kA) + p(eF). Although there is some β-sheet contribution at higher wavelengths (1670–1695 cm^−1^) for p(kG) + p(eF), 17.8%, and p(kA) + p(eF), 8.6%, the band centered around 1625 cm^−1^ is the main contribution to strong β-sheet formation in crystallized proteins [58,59].

**Figure 6:**
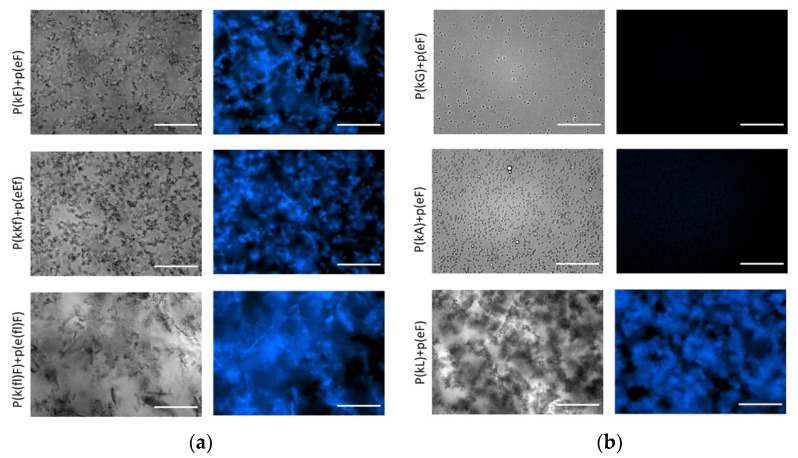
Optical and fluorescence images of complexes with thioflavin T (ThT). The concentration of ThT is 20 μM and all complex solutions were prepared at a total concentration of 5 mM with respect to the monomer charge. (**a**); (**b**) right and left panels show bright field and fluorescence images, respectively. DAPI filter with Ex/Em of 377/447 nm was used for fluorescence imaging. All images were taken under the same conditions. All sequence pairs except p(kG) + p(eF) and p(kA) + p(eF) present fluorescence, indicating β-sheet formation. Scale bars, 50 µm.

The strong β-sheet contribution and also increased hydrophobicity cause p(kL) + p(eF) to form a compact structure with a solid precipitate appearance, while p(kG) + p(eF) and p(kA) + p(eF) form liquid coacervates (Figure 6). Likewise, p(kF) + (eF), p(kKf) + p(eEf), and p(k(fl)F) + p(e(fl)F) all have high β-sheet content at around 1625 cm^−1^, indicating strong interactions between the chains and result in solid complexes as shown in the left-hand side of Figure 6. With the same sequence patterning of alternating chirality, we previously reported that enhanced sidechain length provides more steric hindrance and disrupts hydrogen bonding [11]. Here, we observe that π-interactions and hydrophobic effects can have a dominant role in secondary structure formation. These interactions can promote strong bonding and result in solid complexation.

To further elucidate the interplay between π-interactions and hydrophobic interactions, we replaced p(eF) with the fluorinated polyanion, p(e(fl)F) in sequence pairing with the p(kL). The FTIR spectrum of p(kL) + p(e(fl)F) is shown in Figure 5b and the secondary structure analysis is shown in the last column of Table 3.

Interestingly, the amount of β-sheet formation increases in p(kL) + p(e(fl)F) complexes compared to p(kL) + p(eF) complexes. We again attribute this increased β-sheet formation to greater hydrophobicity of p(e(fl)F) compared to p(eF). It is worth noting that p(kL) + p(e(fl)F) also contain greater β-sheet content in FTIR analysis than p(k(fl)F) + p(e(fl)F) likely due to the larger side-chain of p(k(fl)F) compared to p(kL), which can disrupt hydrogen bonding, resulting in a less compact structure.

To provide further evidence in support of the observed β-sheet formation in FTIR, we investigated the effect of thioflavin T (ThT) on complexes. ThT is a benzothiazole dye that becomes highly fluorescent when binding to protein aggregates [60]. Once in solution, aromatic rings of ThT, benzylamine, and benzathiole, rotate freely around a carbon-carbon bond that connects them. By this rotation, they can quench the excited states produced by photon excitation. Upon binding to β-sheet sites such as in amyloid fibrils, the rings become immobilized and can maintain the excited state, resulting in high fluorescence intensity [61]. Fluorescence imaging of the complexes in Figure 6 shows increased fluorescence intensity upon mixing of ThT with β-sheet-rich peptide pairs. ThT in aqueous solution shows a very weak fluorescence that was subtracted from all images using ImageJ software. No fluorescence intensity is observed for liquid complexes, p(kG) + p(eF) and p(kA) + p(eF), suggesting insufficient β-sheet formation. The fluorescence intensity of all other sequence pairs confirms fluorescence activation of ThT upon binding to aggregates. The p(kL) + p(e(fl)F) sequence pair also shows fluorescence intensity upon addition of ThT, confirming the presence of β-sheet-rich domains (Supplementary Figure S5).

### 3.4. Stability of Sequence Pairs Against Salt

Turbidity measurements of polypeptide pairs provide qualitative information on the extent of complex formation [62]. Ionic association of oppositely charged polyelectrolytes decreases at increased salt concentrations due to the screening effect of salt on the charged groups [63,64,65]. Therefore, we can examine the interplay between ionic and non-ionic interactions at varied salt concentration and their influence on complex formation using turbidity measurements.

**Figure 7:**
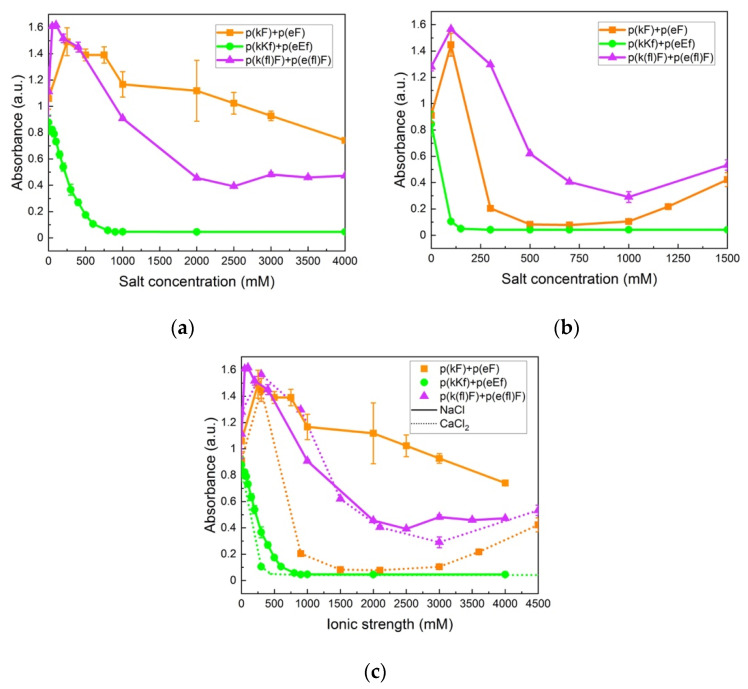
Turbidity measurements of sequence pairs: (**a**) at varied concentrations of sodium chloride (NaCl); (**b**) at varied concentrations of calcium chloride (CaCl_2_); (**c**) at varied ionic strengths of NaCl (solid lines) and CaCl_2_ (dotted lines). High stability is observed for p(kF) + p(eF) and p(k(fl)F) + p(e(fl)F) against both salts. p(kKf) + p(eEf), with higher charge density, is more sensitive to the screening effect of salt, showing a critical salt concentration at 900 and 150 mM for NaCl and CaCl_2_, respectively. All sequence pairs were prepared at equal stoichiometry with a total concentration of 5 mM with respect to the monomer charge.

The change in turbidity at varied sodium chloride (NaCl) concentrations is shown in Figure 7a. At low salt concentrations the turbidity increases for p(kF) + p(eF) and p(k(fl)F) + p(e(fl)F) sequence pairs. This enhanced turbidity can be explained by the higher degree of swelling of complexes or their flocculation at lower ionic strengths [66]. In the low concentration range of salt, the complexes swell and become larger causing a higher scattering intensity [67]. At higher salt concentrations, the turbidity decreases due to the screening effect of salt. Turbidity of p(kKf) + p(eEf) decreases as NaCl concentration increases until it reaches the critical salt concentration at 900 mM, beyond which no phase separation is observed. The screening effect of salt is more pronounced on this sequence pair compared to p(kF) + p(eF) and p(k(fl)F) + p(e(fl)F) due to its higher charge density. Although increased charge density has been shown to increase the amount of complex formation and stability against salt [68,69], the combination of electrostatic, hydrophobic, and π-interactions results in higher stability against salt as shown with both p(kF) + p(eF) and p(k(fl)F) + p(e(fl)F) sequence pairs that have high turbidity values even at 4 M of added salt. Li et al. [70], also observed a turbidity plateau at high salt concentrations, indicative of high salt resistance, for polyacrylic acid and polyallylamine hydrochloride complexes. They attributed this salt stability to non-electrostatic interactions such as hydrophobicity and hydrogen bonding. Lower turbidity values of p(k(fl)F) + p(e(fl)F), at increased NaCl concentrations, compared to p(kF) + p(eF) can be caused by reduced π-interactions as the result of fluorine substitution on the phenylalanine ring. The phase behavior of complexes at examined salt concentrations was also investigated by optical microscopy as shown in Figure 6 in the absence of salt. The p(kF) + p(eF), p(kKf) + p(eEf), and p(k(fl)F) + p(e(fl)F) images all show amorphous solids at all salt conditions (Supplementary Figure S6). Interestingly, no phase transition from solid to liquid occurs with increasing salt in this system, unlike what has been observed for other polyelectrolyte complexes [13,71]. These results coincide with other polypeptide complexes that contain hydrogen bonding, where salt eventually prevents complex formation, but the complexes are always solid [10]. Here the salt stability is even higher suggesting that hydrophobicity and π-interactions stabilize the complexes at high ionic strengths.

We further examined the stability of complexes against a divalent salt, calcium chloride (CaCl_2_). As shown in Figure 7b, the overall turbidity values of complexes in CaCl_2_ are lower at the same concentration compared to NaCl. Comparison of turbidity values at an equivalent ionic strength of salts, as per Figure 7c, also indicates a more inhibitory effect on the complex formation of calcium ions compared to sodium ions. This difference can be explained by the Hofmeister series, where the more chaotropic nature of the calcium ion compared to the sodium ion, tends to promote salting-in behavior and increases protein solubility and unfolding [72,73]. In addition, p(kKf) + p(eEf) has a lower critical salt concentration (150 mM) in CaCl_2_ (confirmed using optical microscopy in Supplementary Figure S6) versus NaCl. The same trend is apparent when comparing ionic strength as seen in Figure 7c. Perry et al. also observed greater efficiency of divalent salts in decreasing complex formation compared to monovalent salts that was explained by the increased solubility of the polyelectrolyte [67]. Interestingly, we observed higher turbidity values for p(k(fl)F) + p(e(fl)F) compared to p(kF) + p(eF) in CaCl_2_. Since later salts in the Hofmeister series weaken hydrophobic interactions, higher turbidity values of (k(fl)F) + p(e(fl)F) can be attributed to its higher hydrophobicity compared to the p(kF) + p(eF) sequence pair and higher stability against CaCl_2_.

**Figure 8:**
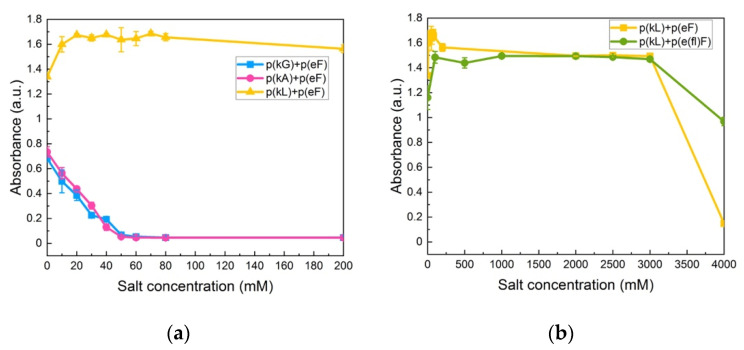
Turbidity measurements at varied NaCl concentrations: (**a**) p(kG), p(kA), and p(kL) sequences paired with p(eF); (**b**) p(kL) + p(eF) and p(kL) + p(e(fl)F). Complexes with p(kL) show high stability against salt. All sequence pairs were prepared at equal stoichiometry with a total concentration of 5 mM with respect to the monomer charge.

Krainer et al. also reported that non-ionic and hydrophobic interactions govern phase separation at high salt concentrations [74]. They found that in the high-salt regime, where electrostatic interactions are no longer involved and screened out, the π-π pairing has a dominant role in stabilizing complexes. Furthermore, they studied the stability of π-involved interactions at high salt concentrations by analyzing cationic and aromatic amino acid pairs. For instance, in the low-salt regime, charged groups of lysine interact with aromatic groups of phenylalanine (cation-π interaction). The interaction then changes at high-salt conditions (3 M NaCl) to the interactions between the methyl groups of lysine and the aromatic rings of phenylalanine (hydrophobic interaction). We also investigated the effect of decreased π-interactions on salt stability by examination of p(kG), p(kA), and p(kL) sequence pairing with the p(eF) sequence at varied NaCl concentrations. As shown in Figure 8a, p(kG) + p(eF) and p(kA) + p(eF) complexes show a critical salt concentration of 60 mM, while p(kL) + p(eF) has a high salt resistance. Bright-field microscopy images of complexes at different salt concentrations, as shown in Supplementary Figure S7, indicate the formation of a soluble phase around 60 mM for p(kG) + p(eF) and p(kA) + p(eF), and the persistent solid phase for p(kL) + p(eF). p(kL) + p(eF) was then compared with the more hydrophobic pair, p(kL) + p(e(fl)F), at higher salt concentrations (Figure 8b). Initially, p(kL) + p(eF) presents somewhat higher turbidity values than p(kL) + p(e(fl)F), followed by a similar trend at higher salt concentrations, and then turbidity decreases more dramatically near 4 M NaCl. This behavior suggests the dominance of hydrophobic interactions at higher salt concentrations, while electrostatic contributions such as charge–charge and cation-π interactions are dominant at lower ionic strengths.

### 3.5. Compartmentalization of a Small Hydrophobic-Charged Molecule Using Sequence Pairs

In order to investigate the effect of the interplay between π and non-π interactions on the compartmentalization behavior of complexes, we selected a model dye for which π-interactions have been shown to promote its partitioning to the complex phase [40]. Methylene blue (MB) is a positively charged molecule that contains aromatic rings. We examined the interplay between interactions on the encapsulation of MB by UV-vis spectroscopy. Complex solutions were prepared for each sequence pair with a total polymer concentration of 5 mM (charge basis) and 100 µM of MB. All sample solutions were kept at room temperature for 24 h and then centrifuged for 30 min at 10,000 pm. The supernatant phases were then carefully removed and dispensed into a 96-well plate for UV-vis measurements.

**Figure 9:**
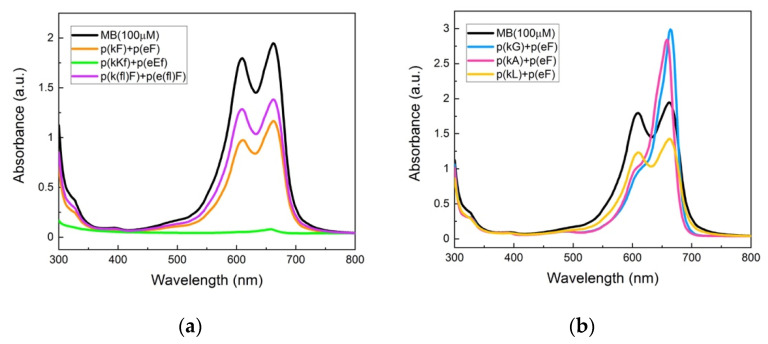
UV-vis absorbance spectroscopy of methylene blue and supernatant solutions: (**a**) p(kF) + p(eF), p(kKf) + p(eEf), and p(k(fl)F) + p(e(fl)F). The p(kKf) + p(eEf) supernatant solution has almost a flat absorbance, indicating complete encapsulation of MB molecules within the complex phase. p(k(fl)F) + p(e(fl)F) has a higher absorbance intensity than p(kF) + p(eF) likely due to reduced π-interactions with MB as the result of the fluorine substitution on the phenylalanine ring; (**b**) p(kG), p(kA), and p(kL) sequences paired with p(eF). The supernatant solution of p(kL) + p(eF) has a lower absorbance intensity compared to p(kG) + p(eF) and p(kA) + p(eF), indicating more encapsulation of MB within the complex phase. p(kL) is more hydrophobic than p(kG) and p(kA) providing stronger interactions with MB. All solutions were prepared with 100 μM of MB and 5 mM of polymers on a monomer charge basis.

UV-vis absorbance spectra of the supernatant phase of complexes are shown in Figure 9a. p(kKf) + p(eEf) with a higher charge density than other sequence pairs shows almost complete encapsulation and a flat absorbance signal of the supernatant solution indicating that all MB molecules are encapsulated within the complex phase. The encapsulation efficiency of the sequence pairs was calculated by deconvolution analysis of the absorbance spectra (Supplementary Figure S8 and Supplementary Table S2). The area under the curve was measured considering both monomer and dimer peaks at 662 nm and 612 nm, respectively. Table 4 contains the calculated encapsulation efficiencies of the sequence pairs. p(kF) + p(eF) has a 42.7% encapsulation efficiency which is higher than encapsulation efficiency of p(k(fl)F) + p(e(fl)F), 27.3%. Both sequence pairs have less charge density than p(kKf) + p(eEf), resulting in less ionic interactions with MB. Moreover, fluorinated sequences have decreased π-interactions due to the presence of fluorine, resulting in even less interaction with MB than the sequence without fluorine. These results suggest the importance of electrostatically-driven π-interactions. Zhao and Zacharia reported similar results in their study on MB partitioning into the complex phases of three different polyanions paired with branched polyethyleneimine (BPEI) [40]. The sequence pair of poly(4-styrenesulfonic acid) (SPS) and BPEI had the highest sequestration of MB compared to other anions, polyacrylic acid, and polyvinyl sulfonate. They explained the higher level of encapsulation efficiency to the cooperative effects of electrostatic and π-π interactions with MB. The aromatic group of SPS engages in π-interactions with the dye and improves MB sequestration into the complex phase.

To further prove the role of π-interactions we examined compartmentalization behavior of p(kG), p(kA), and p(kL) sequence pairs with p(eF) (Figure 9b). Deconvolution analysis of the UV-vis spectra is shown in Supplementary Figure S9 and Supplementary Table S3. As shown in Table 4, the encapsulation efficiency of these complexes is less than the sequence pairs in which both polycation and polyanion have residues with an aromatic ring. The interesting observation with these sequence pairs is the effect of hydrophobicity. As hydrophobicity of the sequence pair increases (p(kG)< p(kA)< p(kL)), encapsulation efficiency increases, highlighting the role of hydrophobic interactions with MB. However, replacement of p(eF) with p(e(fl)F) in the p(kL) + p(eF) sequence pair results in even less encapsulation efficiency, 19.34%, due to the decreased π-interactions with MB (UV-vis spectrum of p(kL) + p(e(fl)F) is compared with p(kL) + p(eF) in Supplementary Figure S10 and deconvolution analysis is shown in Supplementary Figure S9 and Supplementary Table S3). These results confirm the more important cooperative role of ionic and π-interactions in encapsulation of MB than hydrophobic effects.

## 4. Conclusions

The presence of π-containing residues in a sequence pair can affect the secondary structure of polypeptide complexes and their phase behavior. Previous studies have shown the effect of chirality patterns on the phase behavior of polypeptide complexes [10,11,43,44]. Heterochirality disfavors the formation of packed structures due to steric effects and disruption of hydrogen bonding. Here, by using rationally designed π-containing patterns, we demonstrated that despite an alternating pattern of chirality, solid complexes containing hydrogen bonds form, indicating that π-interactions overcome steric hindrance. We attribute the formation of this solid phase to cation-π and π-π interactions in combination with hydrophobic effects. Although electrostatic interactions as long-range forces primarily govern polyelectrolyte complex formation, short-range forces such as hydrophobic and π-interactions increase the stability of complexes [75,76]. At high salt concentrations, electrostatic interactions such as charge-charge and cation-π interactions are screened out, while π-π interactions and hydrophobic effects are less sensitive to the screening effect of salt and drive the complex formation [74]. Sequences with a fluorine substitution on the phenylalanine aromatic ring showed less hydrogen bond formation but still formed solid structures with high stability against salt likely due to increased hydrophobicity. The higher charge density π-containing sequences were able to encapsulate a charged-aromatic small molecule with high efficiency, a strategy that could be used for small molecule therapeutics. However, it is worth noting that the higher charge density π-containing sequences were less stable to salt effects. The incorporation of aromatic amino acids increases the hydrophobicity of the sequences. From our various designs and comparisons, we demonstrated that hydrophobicity plays a significant role in the stability and structure of polyelectrolyte complexes. Overall, here we have demonstrated that by using sequence patterning, we are able to incorporate different features into phase separated complexes that rely on the interplay of the different non-covalent interactions involved. These results could be used to help interpret the driving forces behind protein phase separation and to design vehicles for the delivery of small molecule drugs.

## Figures and Tables

**Table 1:** Sequence pairs of phenylalanine peptides with their measured mass-to-charge ratio, using MALDI-TOF mass spectroscopy. K and E refer to lysine and glutamic acid, respectively. F refers to phenylalanine and (fl)F represents 4-fluoro-phenylalanine. Lower and upper cases are representative of D and L chirality, respectively. All sequences have a degree of polymerization of 30.

Polycations	m/z (g/mol)	Polyanions	m/z (g/mol)
(kF)_15_	4174.24	(eF)_15_	4185.57
(kKfKkF)_5_	4077.47	(eEfEeF)_5_	4097.66
(k(fl)F)_15_	4443.6	(e(fl)F)_15_	4460.08

**Table 2:** Deconvolution analysis of FTIR spectra for sequence pairs. The curve fitting procedure is applied using Origin software. The area under the curves is measured with the Gauss function. Each secondary structure percentage is calculated by dividing the area of its assigned peak by the total area in the 1610–1700 cm^−1^ range. Peaks center around 1610, 1625, 1645, and 1680 cm^−1^ are considered in the secondary structure analysis.

Secondary Structure ^1^	Sequence Pairs		
	p(kF) + p(eF)	p(kKf) + p(eEf)	p(k(fl)F) + p(e(fl)F)
*β-Sheet* (1615–1640 cm^−1^)	73.2%	31.5%	24%
*Random Coil* (1639–1654 cm^−1^)	8.4%	63%	76%
*β-Sheet* (1670–1694 cm^−1^)	18.4% ^2^	5.5%	-

^1^ The secondary structure ranges are adapted from Ref. [54]. ^2^ This signal is centered around 1664 ± 5 cm^−1^.

**Table 3:** Deconvolution analysis of FTIR spectra of sequences p(kG), p(kA), and p(kL) paired with p(eF), and p(kL) paired with p(e(fl)F). The curve fitting procedure is applied using Origin software. The area under the curves is measured with the Gauss function. Each secondary structure percentage is calculated by dividing the area of its assigned peak by the total area in the 1610–1700 cm^−1^ range. Peaks center around 1610, 1625, 1645, and 1680 cm^−1^ are considered in the secondary structure analysis.

Secondary Structure ^1^	Complex Pairs			
	p(kG) + p(eF)	p(kA) + p(eF)	p(kL) + p(eF)	p(kL) + p(e(fl)F)
*β-Sheet* (1615–1640 cm^−1^)	-	-	21.77% ^2^	30.1%
*Random Coil* (1639–1654 cm^−1^)	82.2%	91.4%	78.23%	65.5%
*β-Sheet* (1670–1694 cm^−1^)	17.8%	8.6%	-	4.4%

^1^ The secondary structure ranges adapted from Ref. [54]. ^2^ The value is correlated to two signals, 1615 cm^−1^: 10.33% and 1631 cm^−1^: 11.44%.

**Table 4:** Encapsulation efficiency (EE %) of sequence pairs for methylene blue (MB), calculated based on comparison of area under the absorbance spectra of supernatant solutions with methylene blue solution.

Sequence Pairs	EE (%)	Sequence Pairs	EE (%)
p(kF) + p(eF)	42.7	p(kG) + p(eF)	18.05
p(kKf) + p(eEf)	99.15	p(kA) + p(eF)	21.25
p(k(fl)F) + p(e(fl)F)	27.34	p(kL) + p(eF)	28.83

## Supplementary Materials

The following are available online at https://www.mdpi.com/article/10.3390/polym13132074/s1, Figure S1: MALDI-TOF mass spectroscopy of the peptide sequences, Figure S2: H NMR spectroscopy of the peptide sequences, Figures S3 and S4: Deconvolution analysis of the FTIR spectra of sequence pairs, Figure S5: Optical and fluorescence imaging of p(kL) + p(e(fl)F) with thioflavin T (ThT), Figures S6 and S7: Optical micrographs of sequence pairs at varied salt concentrations, Figures S8 and S9: Deconvolution of the UV-vis spectra of methylene blue in aqueous solution and in the supernatant phase of complexes, Figure S10: UV-vis spectra of MB in aqueous solution and in the supernatant phase of p(kL) + p(eF) and p(kL) + p(e(fl)F), Table S1: Mass-to-charge ratio measurements using MALDI-TOF spectroscopy and the theoretical mass of sequence pairs, Table S2 and Table S3: Deconvolution analysis of UV-vis spectra of methylene blue (MB) in aqueous solution and supernatant solution of sequence pairs.
