# Supplementary material for: Deciphering the Role of π-Interactions in Polyelectrolyte Complexes Using Rationally Designed Peptides
Source: Polymers (Basel). 2021 Jun 24;13(13):2074. doi: 10.3390/polym13132074 (PMC8271475; doi:10.3390/polym13132074)
Supplement: Supplementary file 1 [file polymers-13-02074-s001.zip › polymers-1265208-supplementary_1.pdf]

Supplementary Information

Deciphering the Role of π-Interactions in Polyelectrolyte Complexes using Rationally Designed Peptides

Sara Tabandeh [1], Cristina Lemus [2] and Lorraine Leon [1,3*]

1 Department of Materials Science and Engineering, University of Central Florida, Orlando, FL, USA;
2 Department of Chemistry, University of Central Florida, Orlando, FL, USA;
3 NanoScience Technology Center, University of Central Florida, Orlando, FL, USA;
* Correspondence: lorraine.leon@ucf.edu; Tel.: +1-407-823-5378

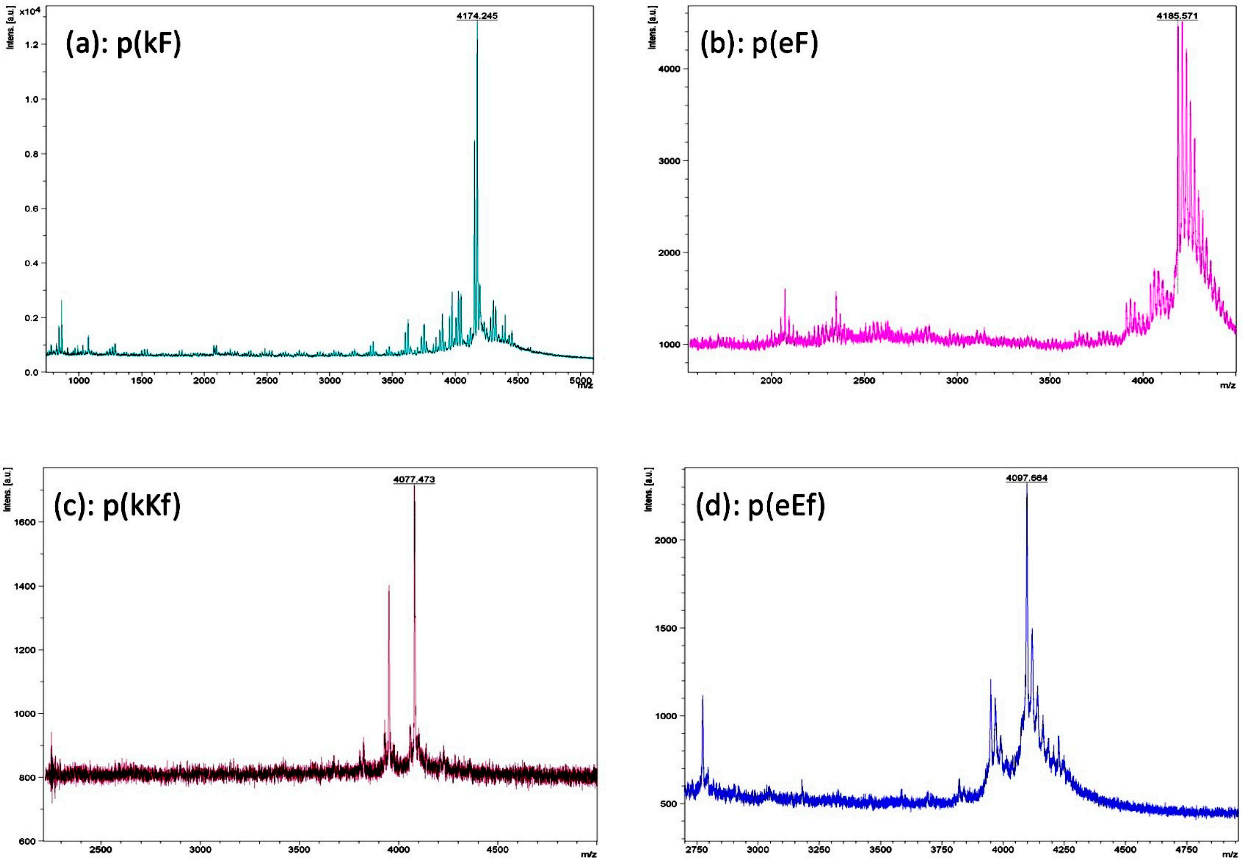

Figure S1. MALDI-TOF mass spectroscopy of the peptide sequences: (a) p(kF); (b) p(eF); (c) p(kKf); (d) p(eEf).

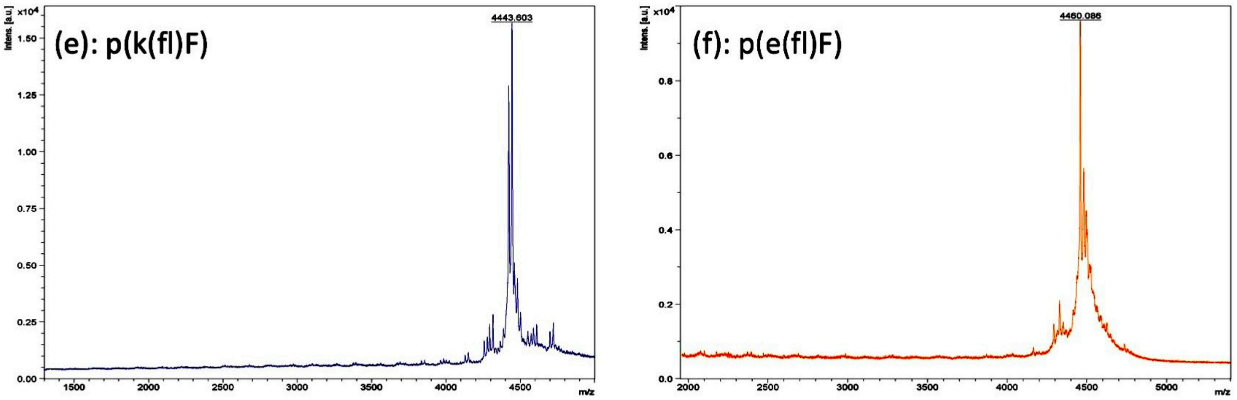

Figure S1. Continued. MALDI-TOF mass spectroscopy of the peptide sequences: (e) p(k(fl)F); (f) p(e(fl)F).

Table S1. Mass-to-charge ratio measurements using MALDI-TOF spectroscopy and the theoretical mass of sequence pairs. K and E refers to lysine and glutamic acid, respectively. F shows phenylalanine and (fl)F represents 4-fluoro-phenylalanine. Lower and upper cases are representative of D and L chirality, respectively.

**Polycations**	**m/z (g/mol)**	**Theoretical mass (g/mol)**	**Polyanions**	**m/z (g/mol)**	**Theoretical mass (g/mol)**
(kF)$_{15}$	4174.24	4147.22	(eF)$_{15}$	4185.57	4161.34
(kKfKkF)$_{5}$	4077.47	4052.21	(eEfEeF)$_{5}$	4097.66	4071.04
(k(fl)F)$_{15}$	4443.6	4417.1	(e(fl)F)$_{15}$	4460.08	4431.2

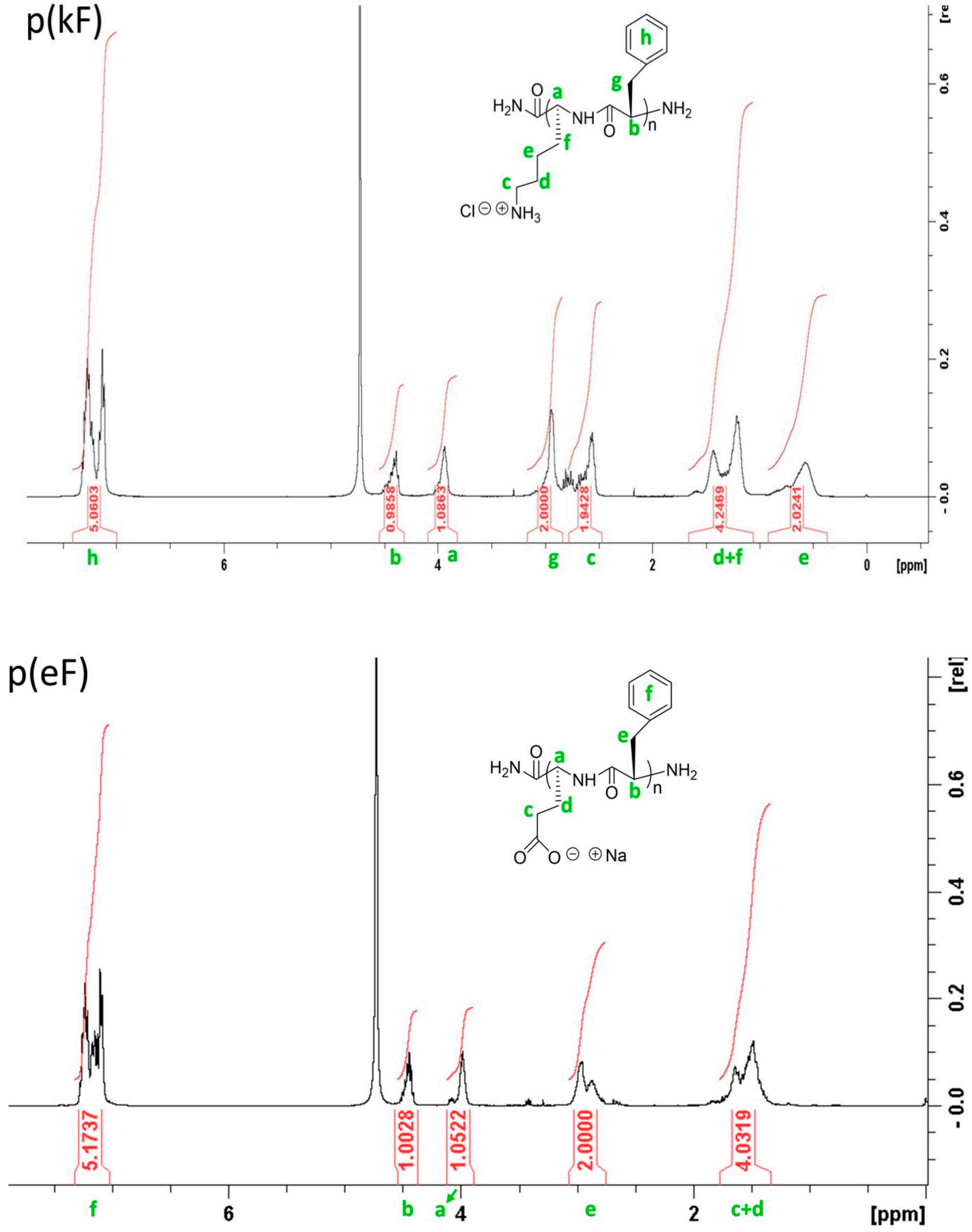

Figure S2. H NMR spectroscopy of the peptide sequences: p(kF) on top; p(eF) on bottom.

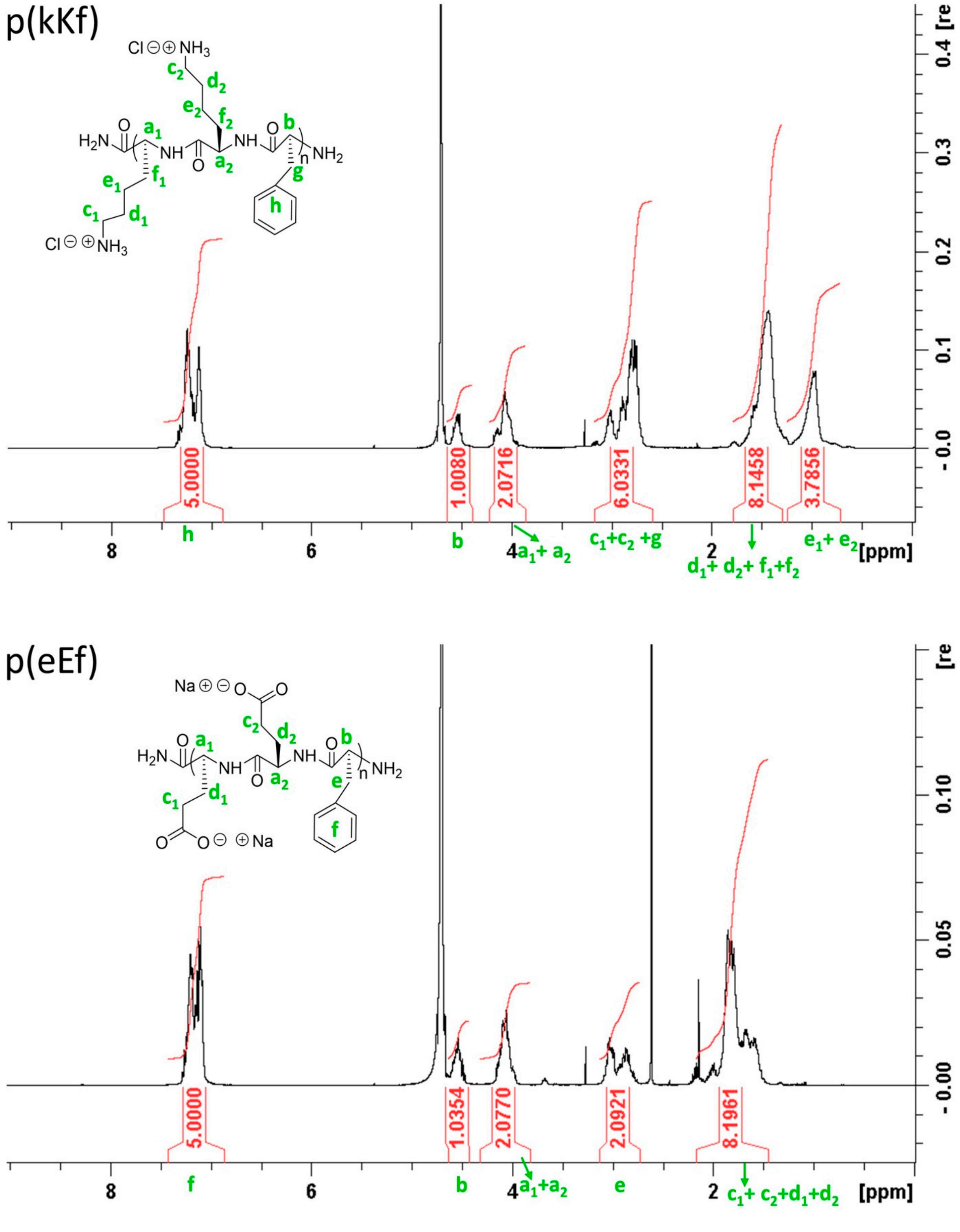

Figure S2. Continued. H NMR spectroscopy of the peptide sequences: p(kKf) on top; p(eEf) on bottom.

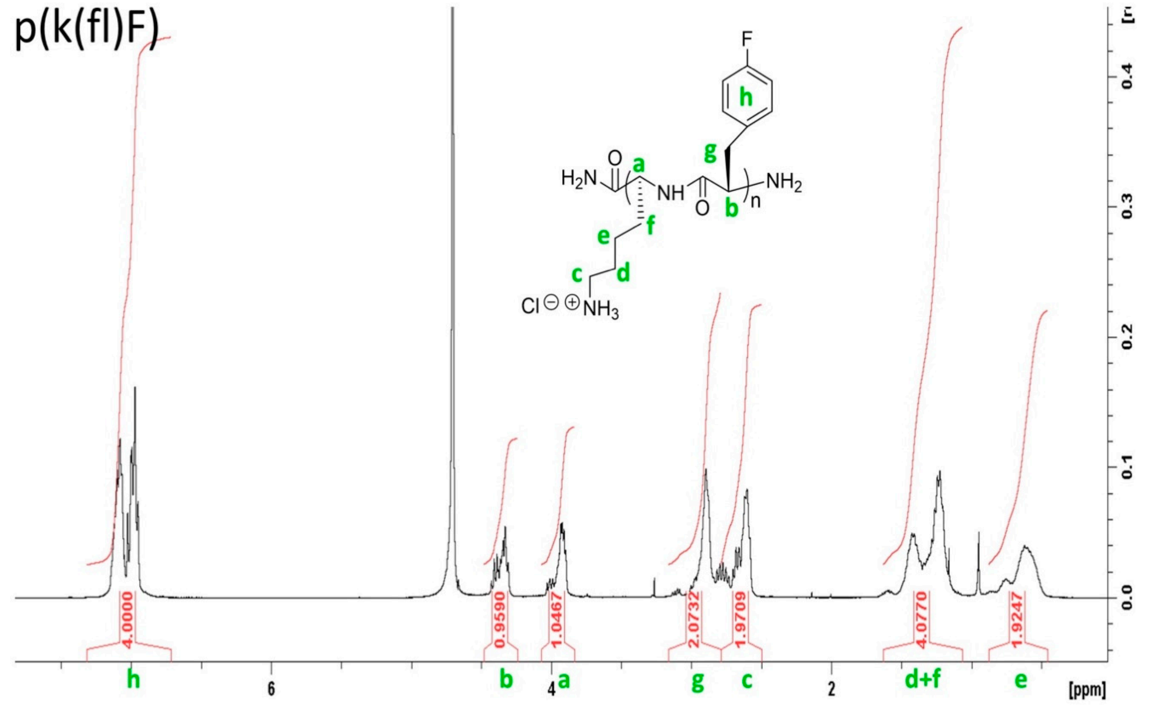

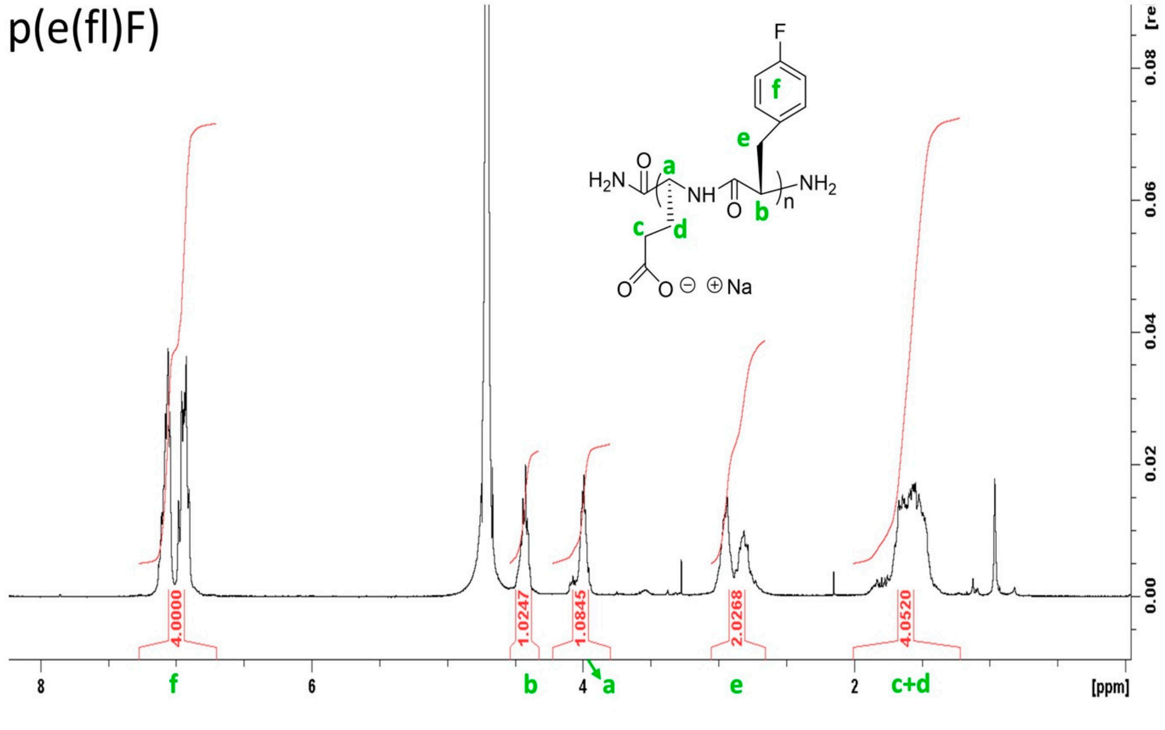

Figure S2. Continued. H NMR spectroscopy of the peptide sequences: p(k(fl)F) on top; p(e(fl)F) on bottom.

(a)

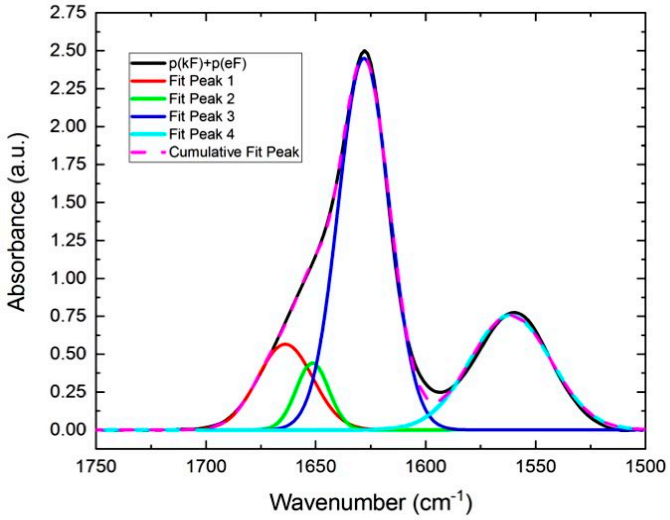

Model	Gauss			
Equation	y=y0 + (A/(w*sqrt(pi/2)))*exp(-2*((x-xc)/w)^2)			
Plot	Peak1(Subtracted_D	Peak2(Subtracted_D	Peak3(Subtracted_D	Peak4(Subtracted_D
y0	6.00832E-4 ± 0.0040	6.00832E-4 ± 0.0040	6.00832E-4 ± 0.0040	6.00832E-4 ± 0.0040
xc	1663.91261 ± 4.9707	1651.50362 ± 0.6854	1628.06371 ± 0.1881	1561.43812 ± 0.1962
w	25.21832 ± 4.11307	14.86012 ± 2.61845	23.27326 ± 0.24485	36.65928 ± 0.47028
A	17.86613 ± 6.98907	8.19766 ± 6.08477	71.4268 ± 1.11909	34.819 ± 0.49278
Reduced Chi-	7.27925E-4			
R-Square (C	0.99836			
Adj. R-Squar	0.99824			

(b)

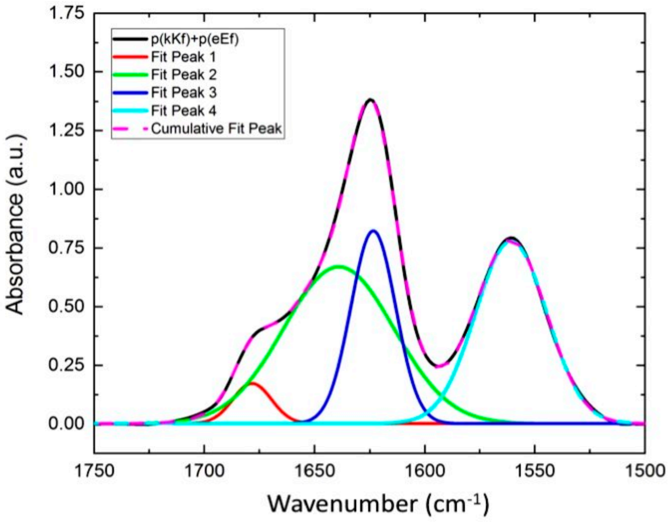

Model	Gauss			
Equation	y=y0 + (A/(w*sqrt(pi/2)))*exp(-2*((x-xc)/w)^2)			
Plot	Peak1(kKf-eEf)	Peak2(kKf-eEf)	Peak3(kKf-eEf)	Peak4(kKf-eEf)
y0	0.00131 ± 9.73405E-4	0.00131 ± 9.73405E-4	0.00131 ± 9.73405E-4	0.00131 ± 9.73405E-4
xc	1678.28872 ± 0.1623	1638.99106 ± 0.43008	1623.38602 ± 0.05508	1560.73202 ± 0.05283
w	17.39928 ± 0.61039	50.06638 ± 0.56668	20.35881 ± 0.1679	32.30035 ± 0.11489
A	3.73975 ± 0.27194	41.96808 ± 0.33379	20.98087 ± 0.42499	31.54948 ± 0.11605
Reduced Chi-Sqr	3.68964E-5			
R-Square (COD)	0.99976			
Adj. R-Square	0.99975			

Figure S3. Deconvolution analysis of the FTIR spectra of sequence pairs: (a) p(kF)+p(eF); (b) p(kKf)+p(eEf).

(c)

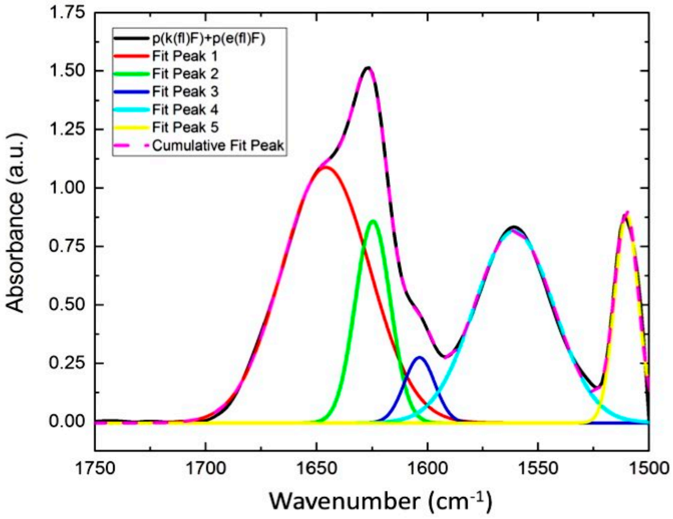

Model	Gauss				
Equation	y=y0 + (A/(w*sqrt(pi/2)))*exp(-2*((x-xc)/w)^2)				
Plot	Peak1(B)	Peak2(B)	Peak3(B)	Peak4(B)	Peak5(B)
y0	-0.00566 ± 0.00295	-0.00566 ± 0.00295	-0.00566 ± 0.00295	-0.00566 ± 0.00295	-0.00566 ± 0.00295
xc	1645.80644 ± 0.59	1624.62544 ± 0.141	1603.56547 ± 0.41	1560.82213 ± 0.130	1510.11041 ± 0.059
w	39.54028 ± 0.7707	15.79867 ± 0.47961	13.86388 ± 0.7885	35.86941 ± 0.31727	10.73307 ± 0.12694
A	54.24409 ± 1.5229	17.10645 ± 1.15945	4.87106 ± 0.37774	37.02621 ± 0.33381	12.0056 ± 0.13519
Reduced Chi-S	3.07598E-4				
R-Square (CO	0.99845				
Adj. R-Square	0.99831				

Figure S3. Continued. Deconvolution analysis of the FTIR spectra of sequence pairs: (c) p(k(fl)F)+p(e(fl)F).

(a)

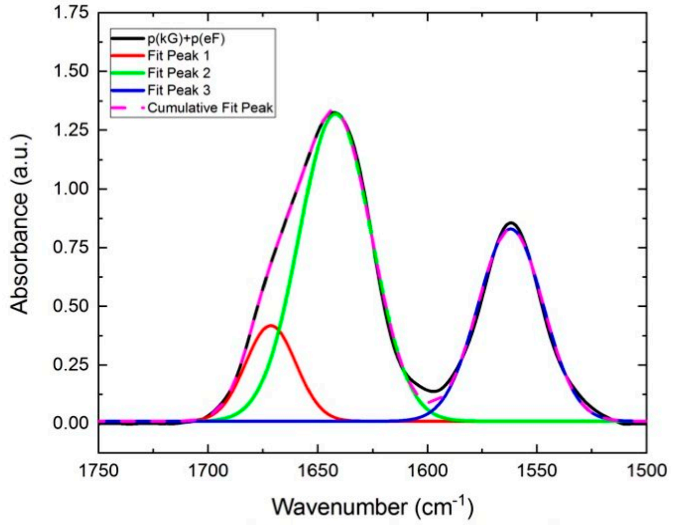

Model	Gauss		
Equation	y=y0 + (A/(w*sqrt(pi/2)))*exp(-2*((x-xc)/w)^2)		
Plot	Peak1(Subtracted_Data Y1)	Peak2(Subtracted_Data Y1)	Peak3(Subtracted_Data Y1)
y0	0.0098 ± 0.00257	0.0098 ± 0.00257	0.0098 ± 0.00257
xc	1671.38892 ± 0.66241	1642.01808 ± 0.36308	1561.97844 ± 0.11296
w	22.87895 ± 0.86115	32.94812 ± 0.48197	29.74486 ± 0.25756
A	11.69357 ± 1.04383	54.09945 ± 1.08405	30.57452 ± 0.28098
Reduced Chi-Sqr	3.56696E-4		
R-Square (COD)	0.99803		
Adj. R-Square	0.99793		

(b)

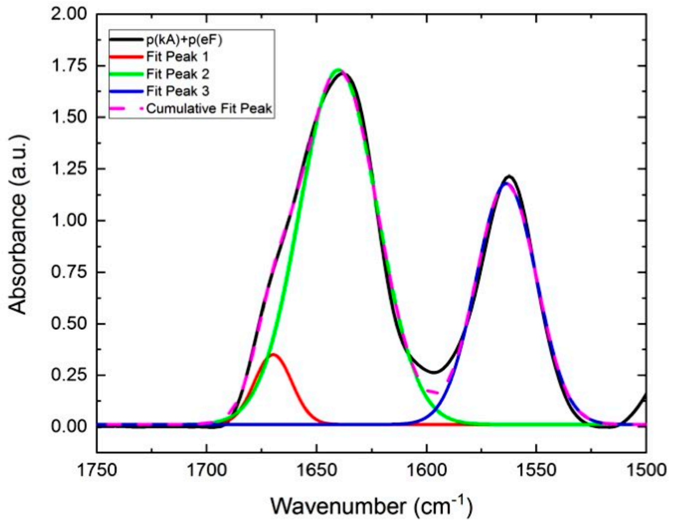

Model	Gauss		
Equation	y=y0 + (A/(w*sqrt(pi/2)))*exp(-2*((x-xc)/w)^2)		
Plot	Peak1(Subtracted_Data	Peak2(Subtracted_Data	Peak3(Subtracted_Data
y0	0.01094 ± 0.00548	0.01094 ± 0.00548	0.01094 ± 0.00548
xc	1669.73781 ± 0.73143	1640.0313 ± 0.3659	1563.61066 ± 0.17532
w	17.02292 ± 1.57748	35.37954 ± 0.64994	27.52006 ± 0.38782
A	7.23435 ± 1.23582	76.18935 ± 1.47407	40.29908 ± 0.58329
Reduced Chi-S	0.00187		
R-Square (CO	0.99406		
Adj. R-Square	0.99374		

Figure S4. Deconvolution analysis of the FTIR spectra of sequence pairs: (a) p(kG)+p(eF); (b) p(kA)+p(eF).

(c)

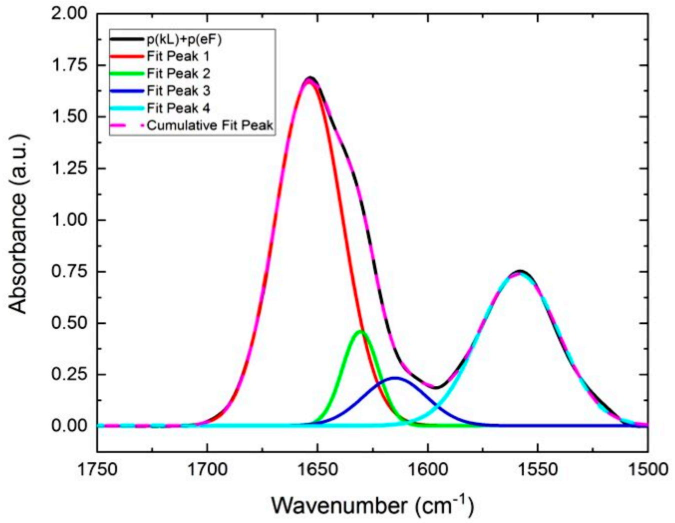

Model	Gauss			
Equation	y=y0 + (A/(w*sqrt(pi/2)))*exp(-2*((x-xc)/w)^2)			
Plot	Peak1(Subtracted_D	Peak2(Subtracted_D	Peak3(Subtracted_D	Peak4(Subtracted_D
y0	0.00169 ± 0.00122	0.00169 ± 0.00122	0.00169 ± 0.00122	0.00169 ± 0.00122
xc	1653.88653 ± 0.142	1630.61949 ± 0.220	1614.90611 ± 3.393	1558.56844 ± 0.102
w	30.48472 ± 0.17439	16.25656 ± 0.70456	28.96307 ± 3.69549	36.60387 ± 0.2229
A	63.62833 ± 0.59346	9.33123 ± 1.56434	8.40842 ± 1.97828	33.86951 ± 0.21007
Reduced Chi	6.96646E-5			
R-Square (C	0.99974			
Adj. R-Squar	0.99972			

(d)

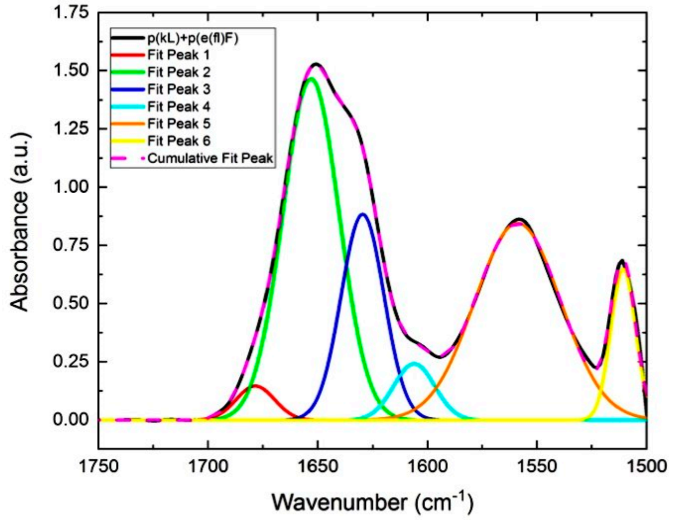

Model	Gauss					
Equation	y=y0 + (A/(w*sqrt(pi/2)))*exp(-2*((x-xc)/w)^2)					
Plot	Peak1(Subtracted_	Peak2(Subtracted_	Peak3(Subtracted_	Peak4(Subtracted_	Peak5(Subtracted_	Peak6(Subtracted_
y0	-0.00157 ± 0.00208	-0.00157 ± 0.00208	-0.00157 ± 0.00208	-0.00157 ± 0.00208	-0.00157 ± 0.00208	-0.00157 ± 0.00208
xc	1678.40799 ± 2.44	1653.09634 ± 0.76	1629.59367 ± 0.86	1606.03952 ± 1.30	1558.79823 ± 0.10	1510.64028 ± 0.05
w	17.38733 ± 2.4091	25.67835 ± 2.3417	19.5602 ± 1.51966	18.72926 ± 1.6024	39.10723 ± 0.2740	11.24318 ± 0.1280
A	3.2014 ± 1.69556	47.17189 ± 5.5757	21.70694 ± 4.6811	5.69398 ± 0.90099	41.53555 ± 0.2818	9.16533 ± 0.10215
Reduced Chi	1.5034E-4					
R-Square (C	0.99934					
Adj. R-Squar	0.99926					

Figure S4. Continued. Deconvolution analysis of the FTIR spectra of sequence pairs: (c) p(kL)+p(eF); (d) p(kL)+p(e(fl)F).

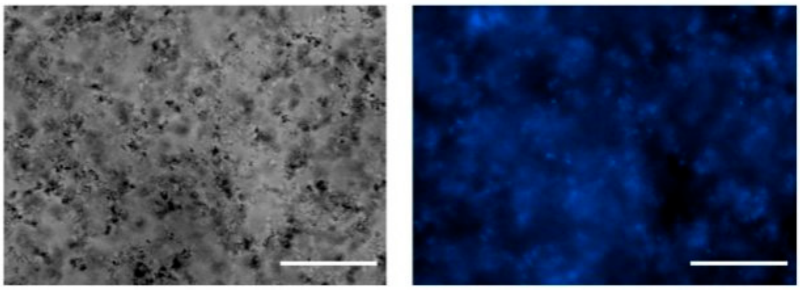

Figure S5. Optical and fluorescence imaging of p(kL)+p(e(fl)F) with thioflavin T (ThT). Scale bars, 50 μm.

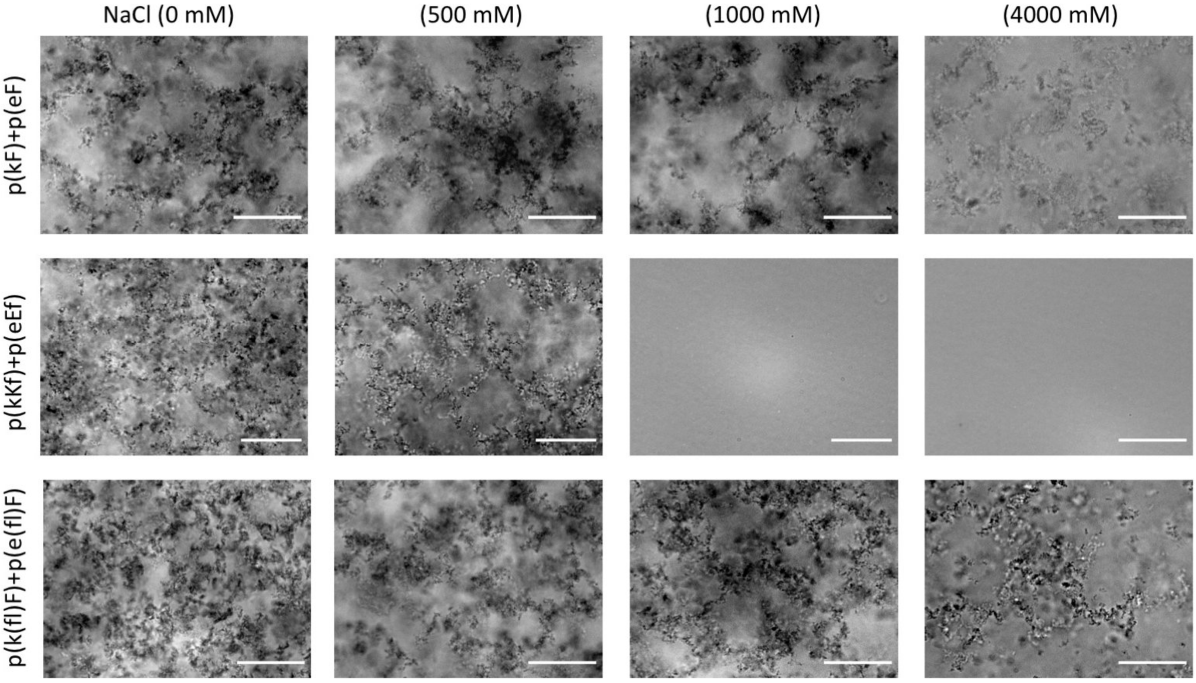

Figure S6. Optical micrographs (bright-field) of sequence pairs at varied salt (NaCl) concentrations. Scale bars, 50 μm.

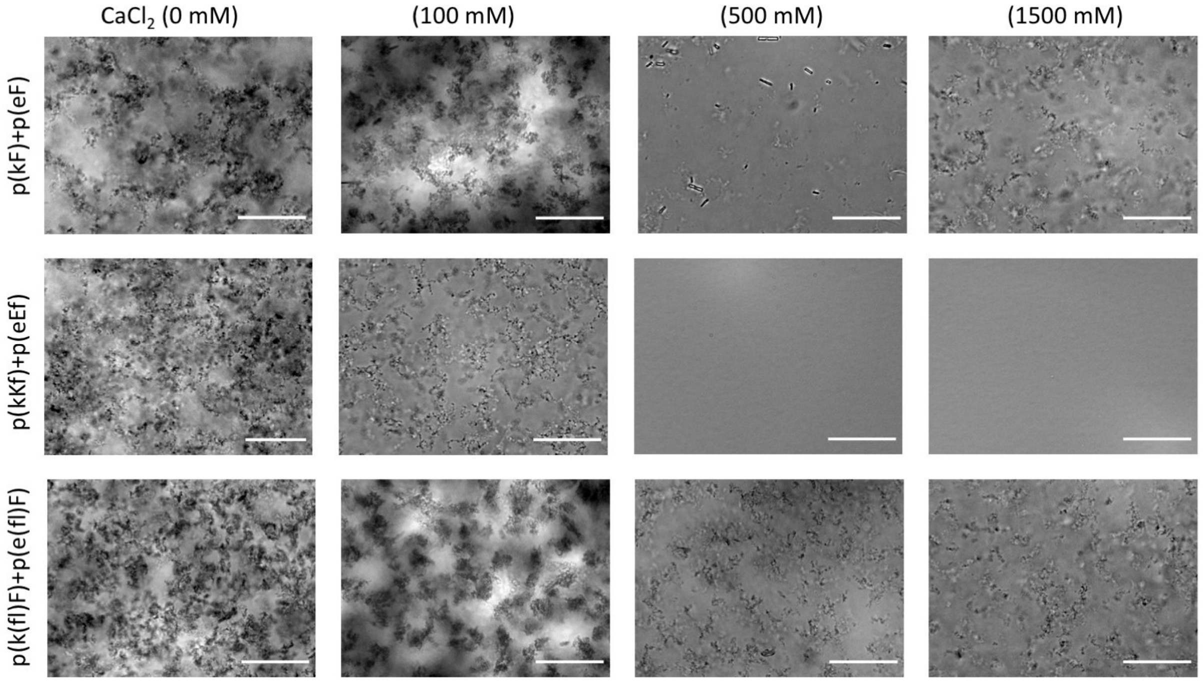

Figure S6. Continued. Optical micrographs (bright-field) of sequence pairs at varied salt ($CaCl_2$) concentrations. Scale bars, 50 μm.

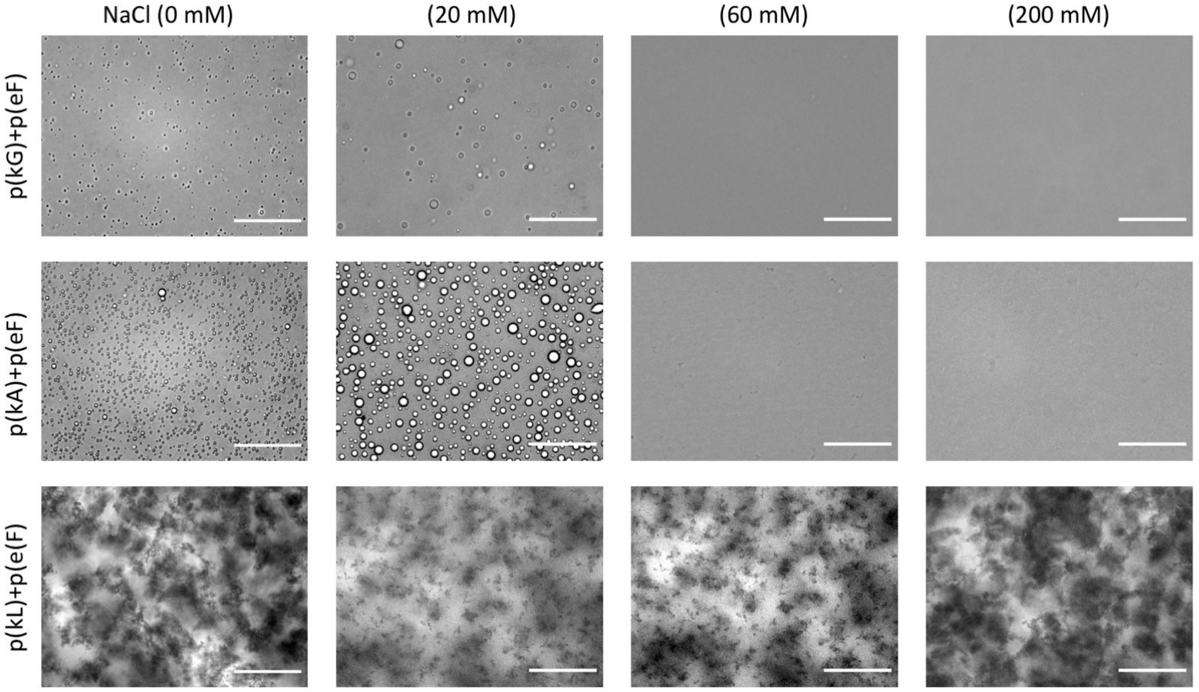

Figure S7. Optical micrographs (bright-field) of sequence pairs at varied salt (NaCl) concentrations. Scale bars, 50 μm.

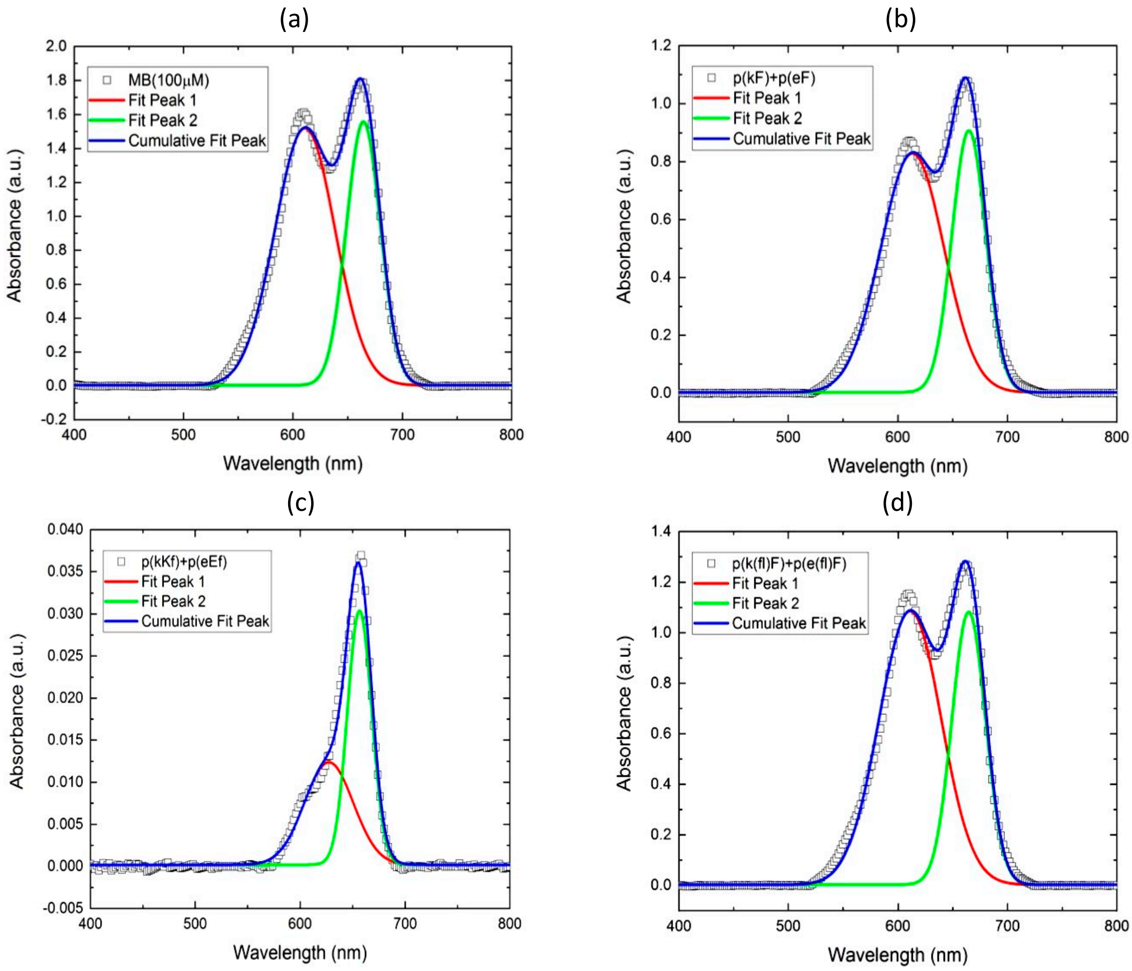

Figure S8. Deconvolution of the UV-vis spectra of methylene blue in aqueous solution and in the supernatant phase of complexes: (a) Methylene blue solution (MB); (b) p(kF)+p(eF); (c) p(kKf)+p(eEf); (d) p(k(fl)F)+p(e(fl)F).

Table S2. Deconvolution analysis of UV-vis spectra of methylene blue (MB) in aqueous solution and supernatant solution of sequence pairs.

**Deconvolution results**	**MB**	**p(kF)+p(eF)**	**p(kKf)+p(eEf)**	**p(k(fl)F)+p(e(fl)F)**
Area at around 612 nm	104.32	59.6	0.71	76.9
Area at around 662 nm	60.41	35	0.87	41.7
Total area for monomers	269.05	154.2	2.29	195.5

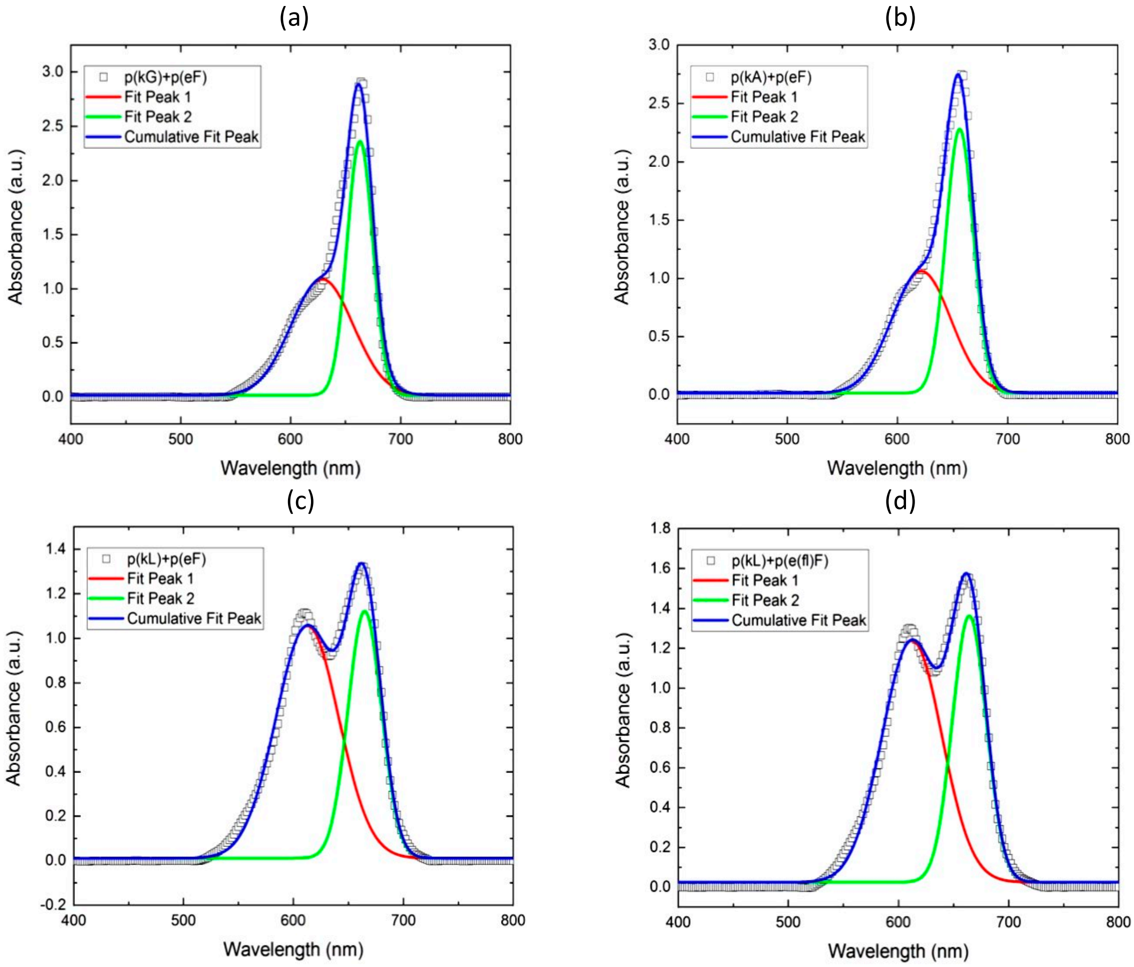

Figure S9. Deconvolution of the UV-vis spectra of methylene blue in aqueous solution and in the supernatant phase of complexes: (a) p(kG)+p(eF); (b) p(kA)+p(eF); (c) p(kL)+p(eF); (d) p(kL)+p(e(fl)F).

Table S3. Deconvolution analysis of UV-vis spectra of methylene blue (MB) in aqueous solution and supernatant solution of sequence pairs.

**Deconvolution results**	**p(kG)+p(eF)**	**p(kA)+p(eF)**	**p(kL)+p(eF)**	**p(kL)+p(e(fl)F)**
Area at around 612 nm	76	70.44	74.55	82.64
Area at around 662 nm	68.48	71	42.38	51.72
Total area for monomers	220.48	211.88	191.48	217

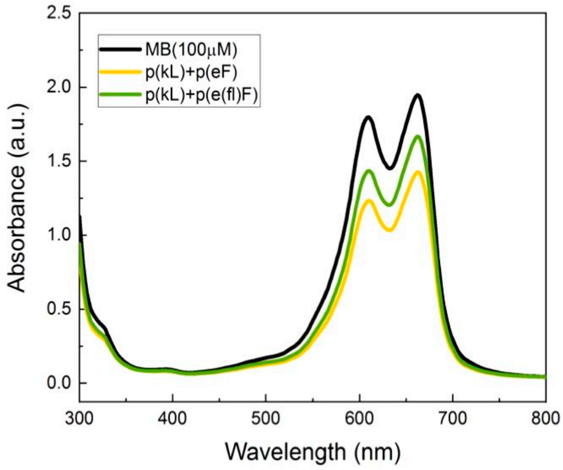

Figure S10. UV-vis spectra of MB in aqueous solution and in the supernatant phase of p(kL)+p(eF) and p(kL)+p(e(fl)F).
