# Supplementary figures and images for: Deciphering the Role of π-Interactions in Polyelectrolyte Complexes Using Rationally Designed Peptides
Source: Polymers (Basel). 2021 Jun 24;13(13):2074. doi: 10.3390/polym13132074 (PMC8271475; doi:10.3390/polym13132074)

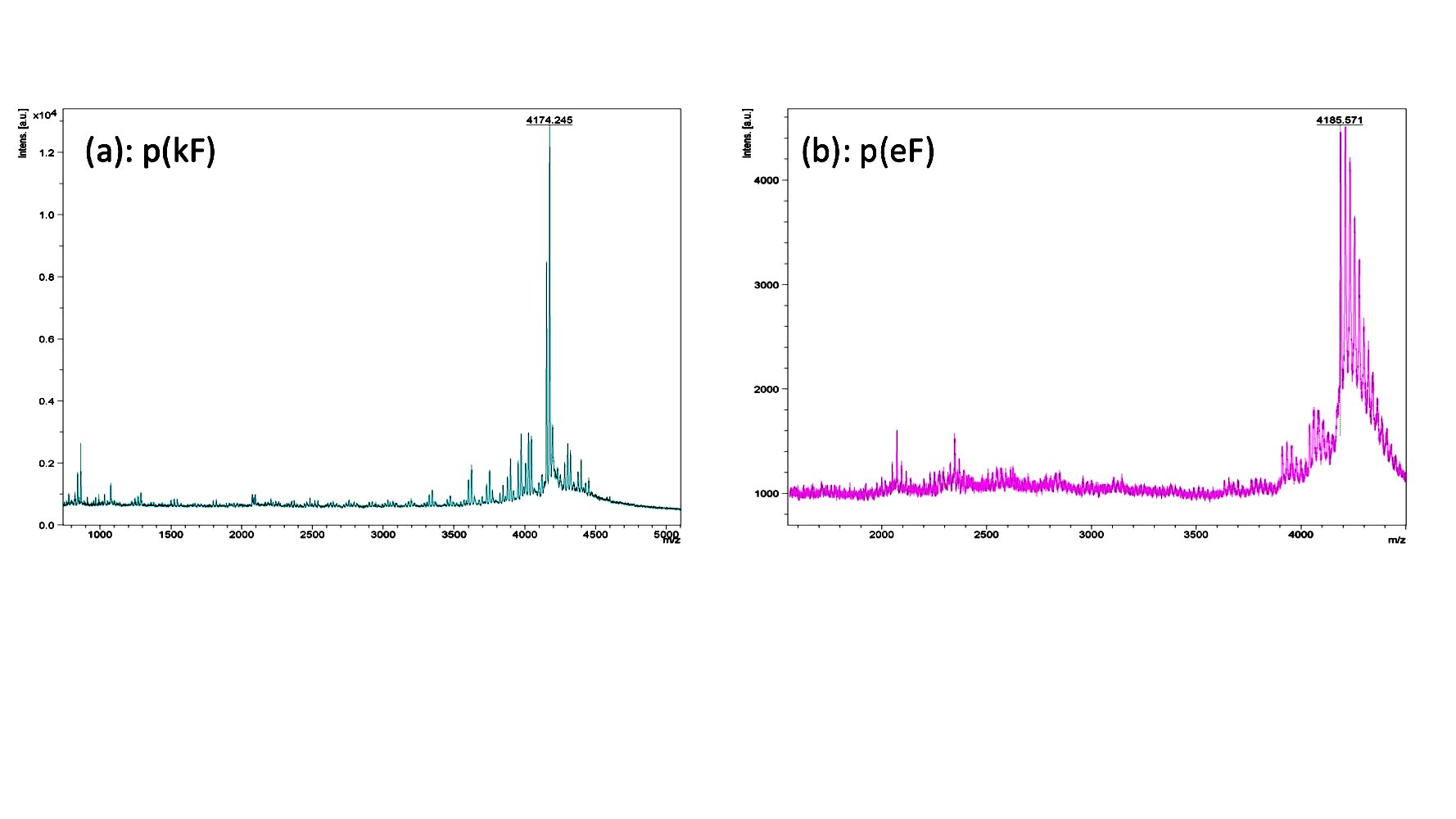

Supplement: Supplementary file 1 [file polymers-13-02074-s001.zip › Suppl. Figures/Figure S1. Maldi-Tof mass spectroscopy of the peptide sequences. (a) and (b).jpg]

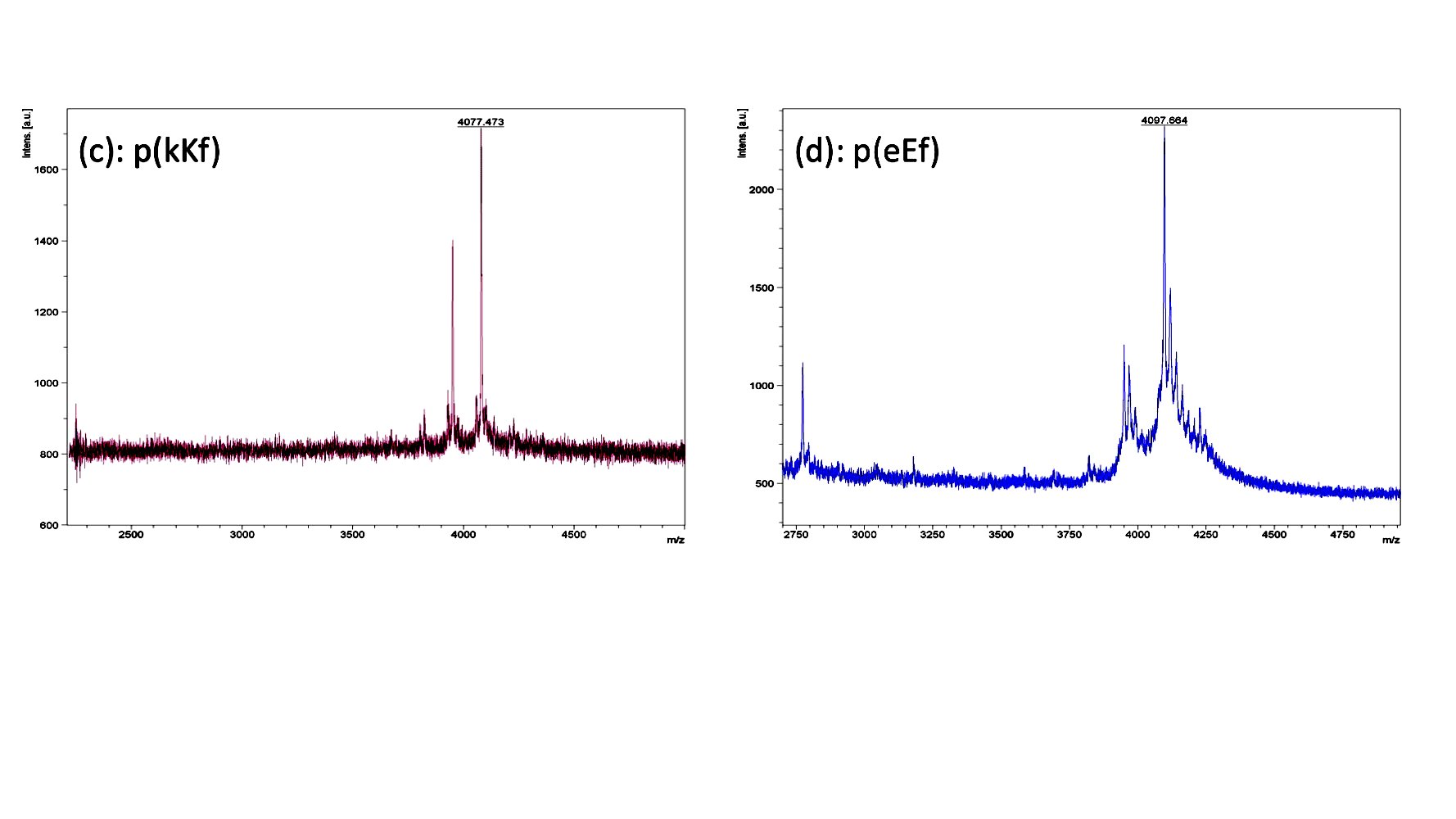

Supplement: Supplementary file 1 [file polymers-13-02074-s001.zip › Suppl. Figures/Figure S1. Maldi-Tof mass spectroscopy of the peptide sequences. (c) and (d).jpg]

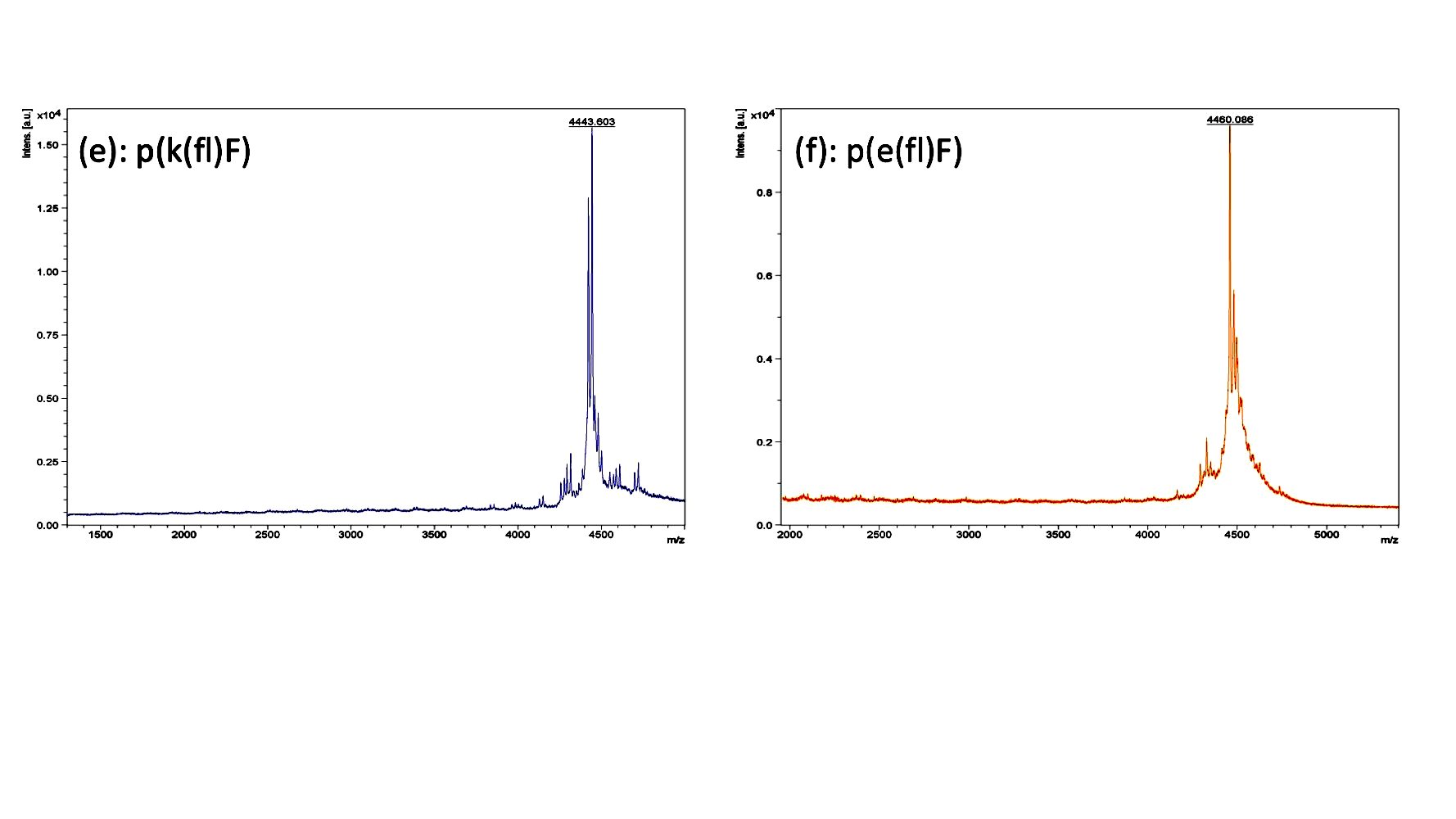

Supplement: Supplementary file 1 [file polymers-13-02074-s001.zip › Suppl. Figures/Figure S1. Maldi-Tof mass spectroscopy of the peptide sequences. (e) and (f).jpg]

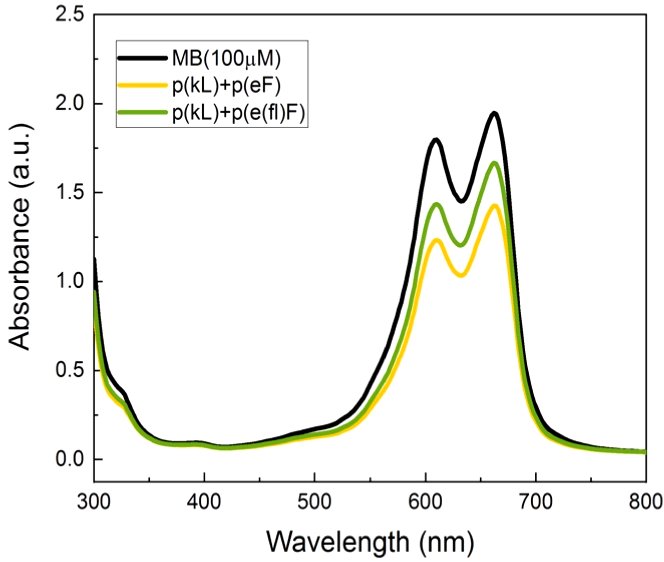

Supplement: Supplementary file 1 [file polymers-13-02074-s001.zip › Suppl. Figures/Figure S10. UV-vis spectra of MB in aqueous solution and in the supernatant phase of p(kL)+p(eF) and p(kL)+p(e(fl)F).jpg]

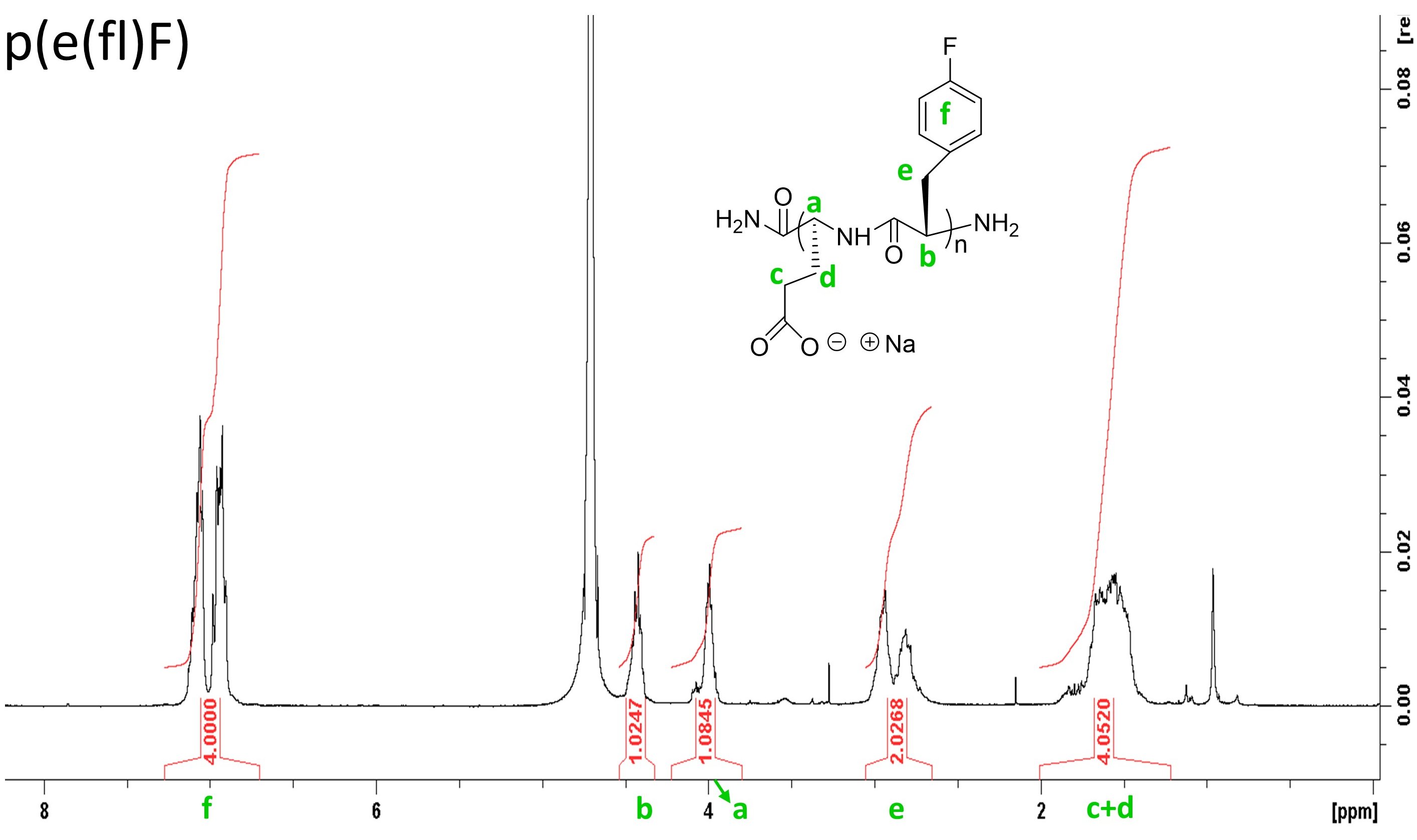

Supplement: Supplementary file 1 [file polymers-13-02074-s001.zip › Suppl. Figures/Figure S2. H NMR spectrum of p(e(fl)F).jpg]

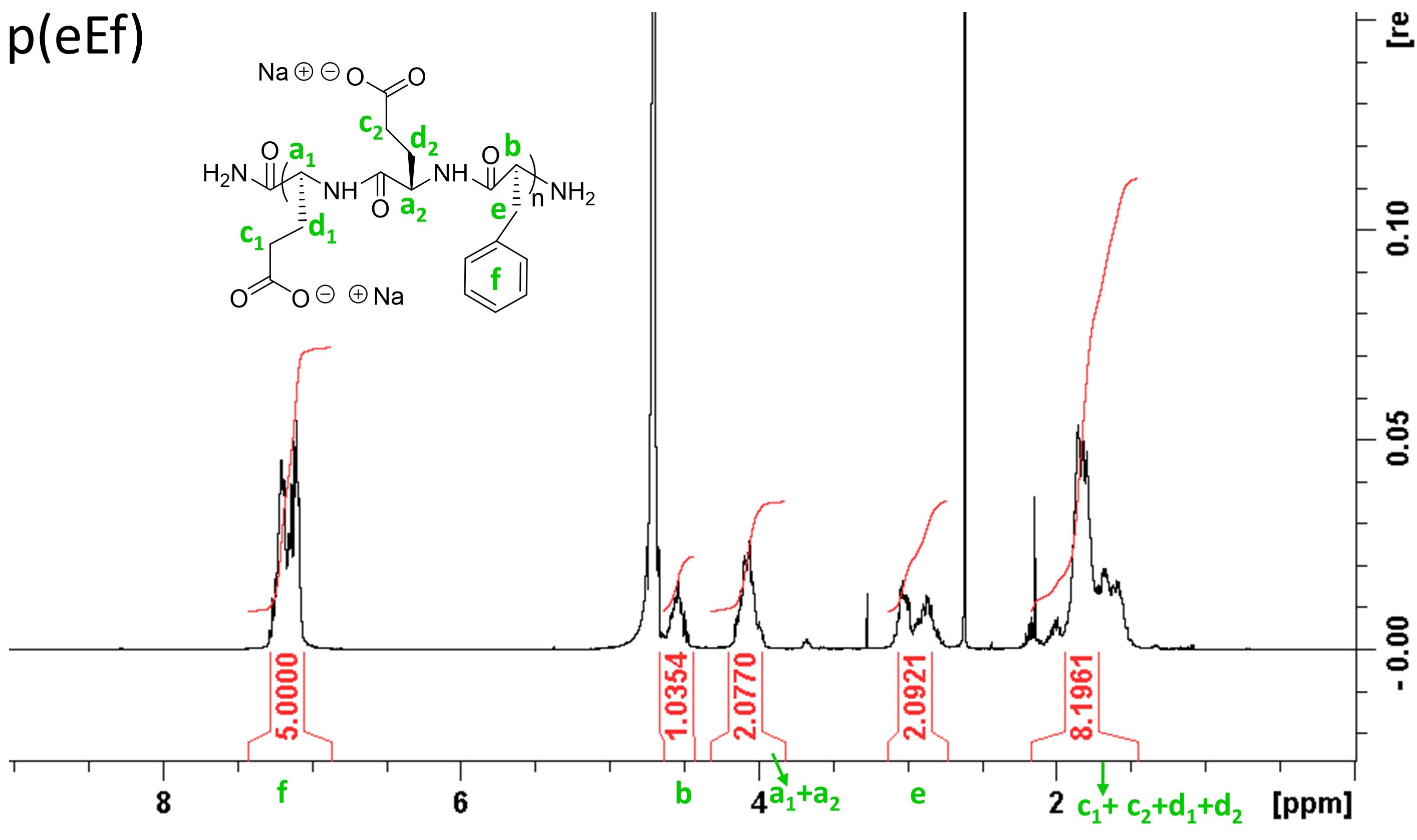

Supplement: Supplementary file 1 [file polymers-13-02074-s001.zip › Suppl. Figures/Figure S2. H NMR spectrum of p(eEf).jpg]

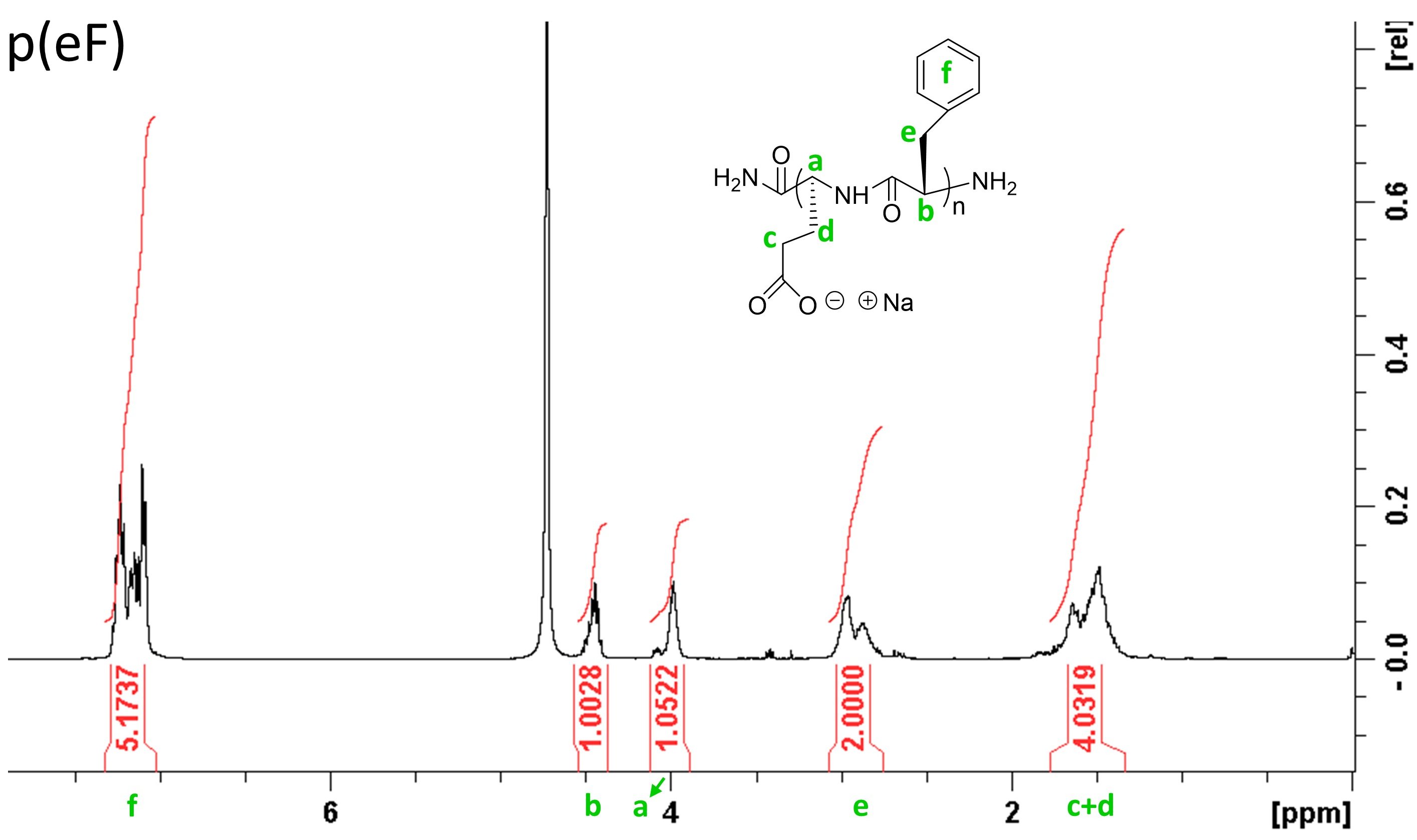

Supplement: Supplementary file 1 [file polymers-13-02074-s001.zip › Suppl. Figures/Figure S2. H NMR spectrum of p(eF).jpg]

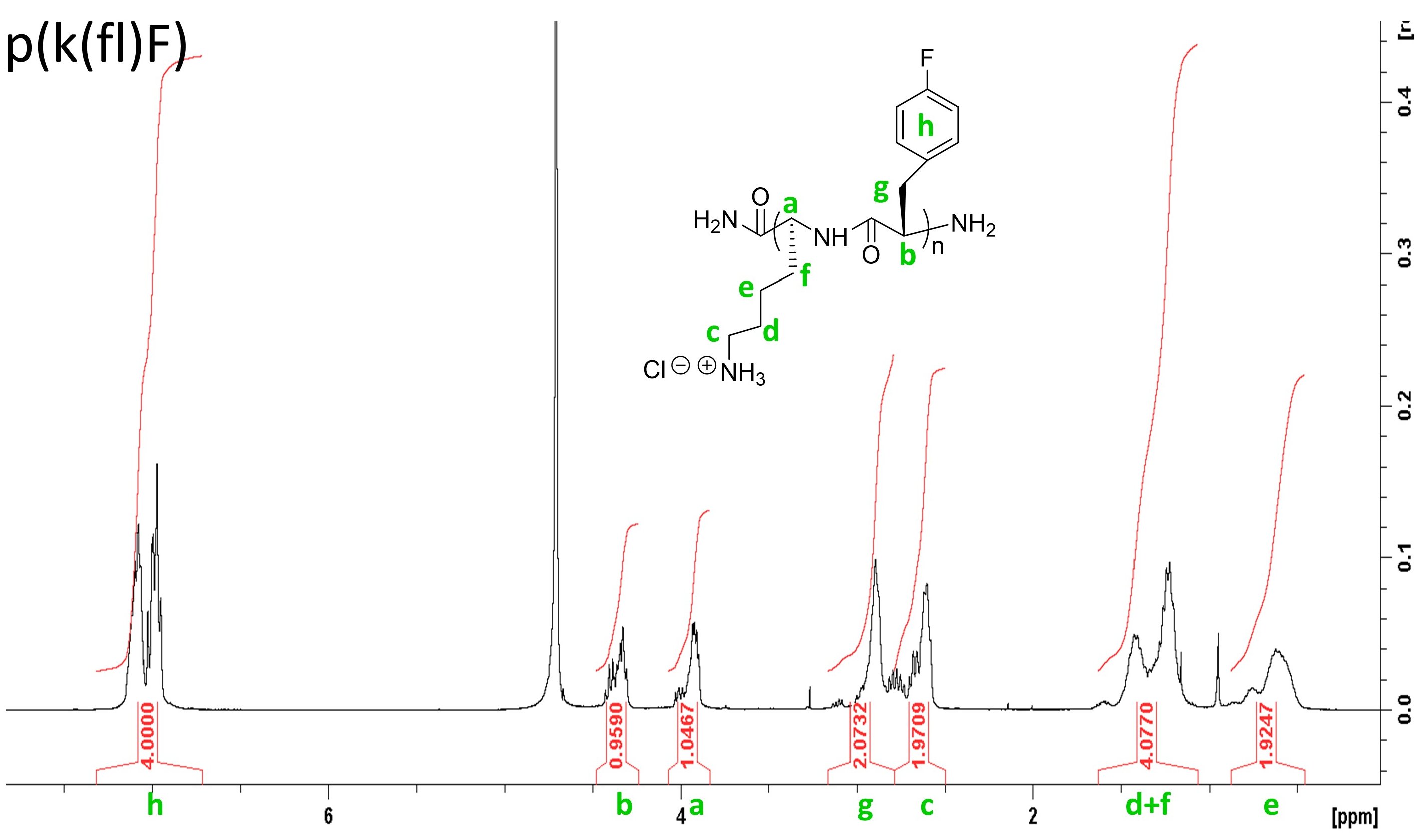

Supplement: Supplementary file 1 [file polymers-13-02074-s001.zip › Suppl. Figures/Figure S2. H NMR spectrum of p(k(fl)F).jpg]

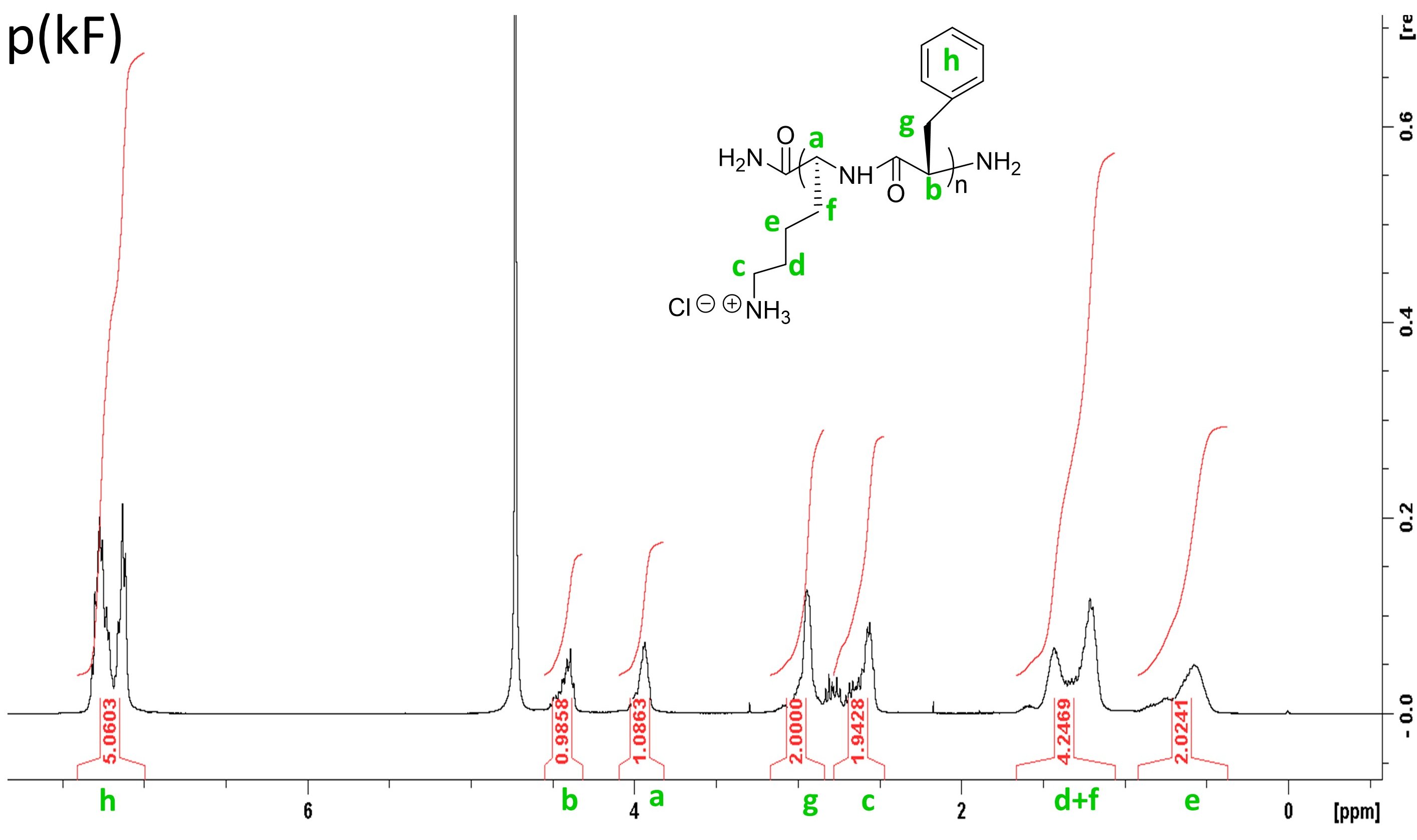

Supplement: Supplementary file 1 [file polymers-13-02074-s001.zip › Suppl. Figures/Figure S2. H NMR spectrum of p(kF).jpg]

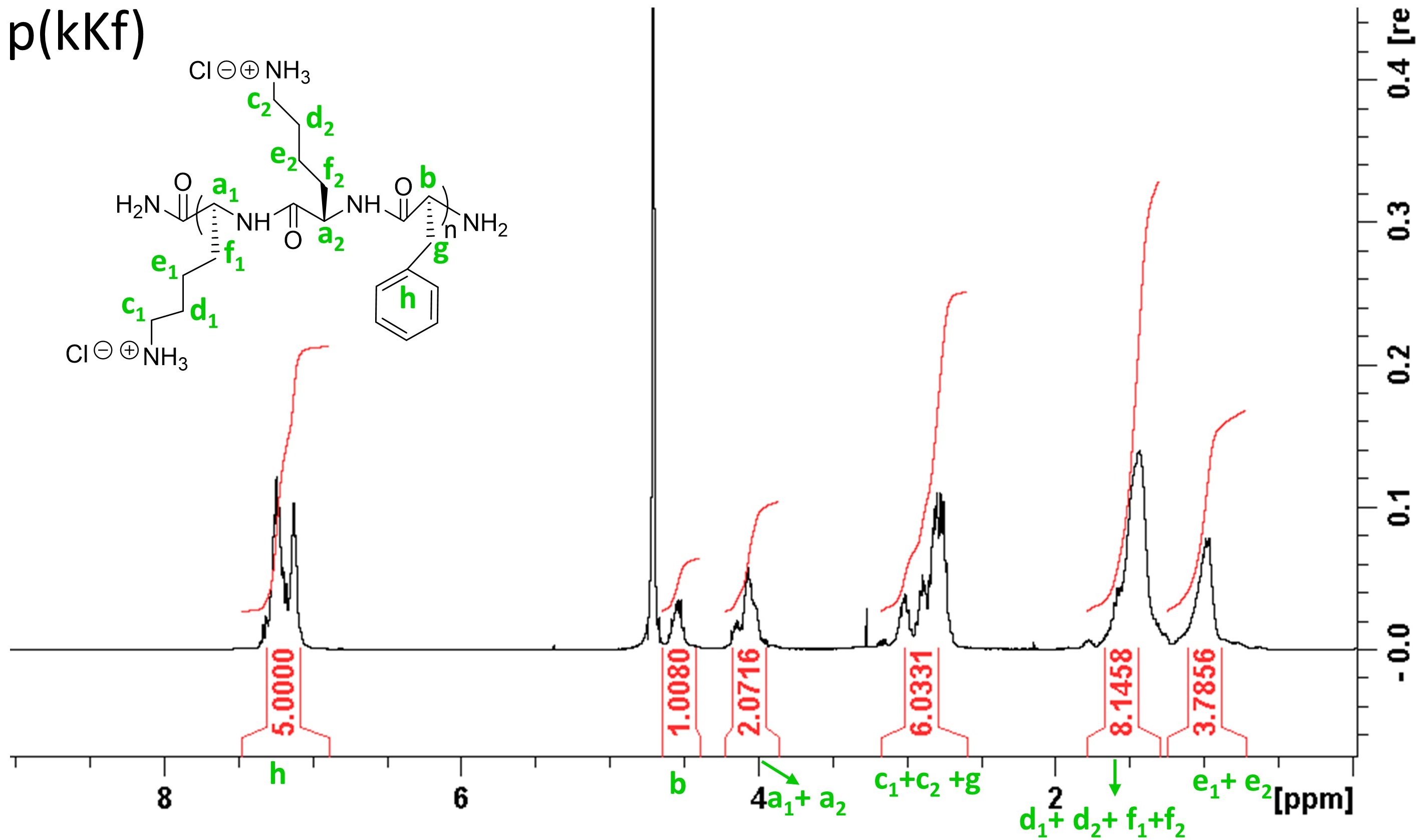

Supplement: Supplementary file 1 [file polymers-13-02074-s001.zip › Suppl. Figures/Figure S2. H NMR spectrum of p(kKf).jpg]

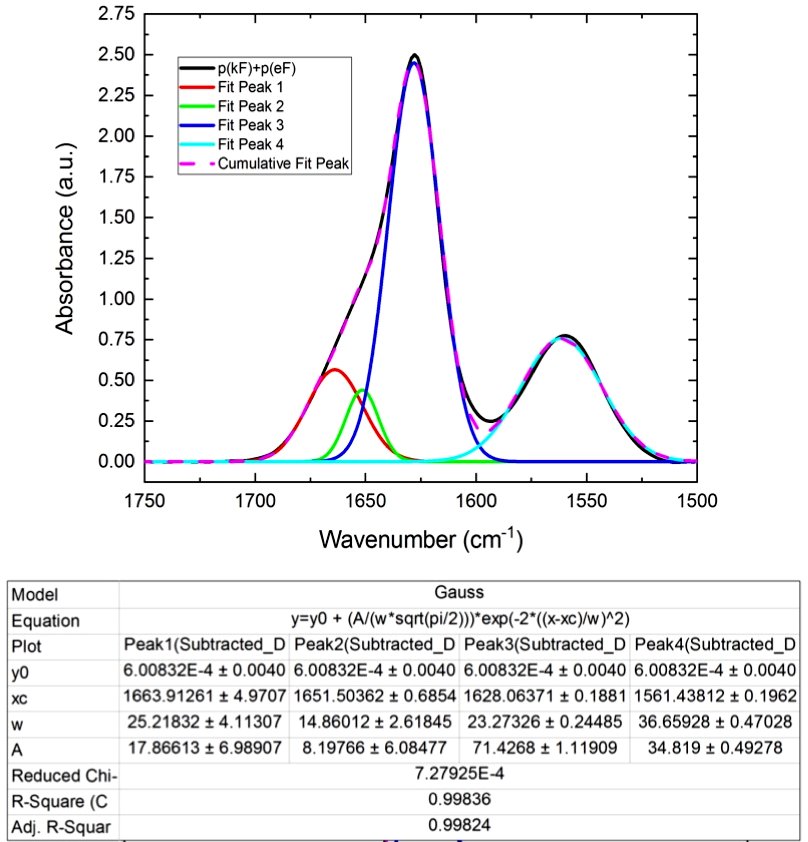

Supplement: Supplementary file 1 [file polymers-13-02074-s001.zip › Suppl. Figures/Figure S3. Deconvolution analysis of the FTIR spectra of sequence pairs (a).jpg]

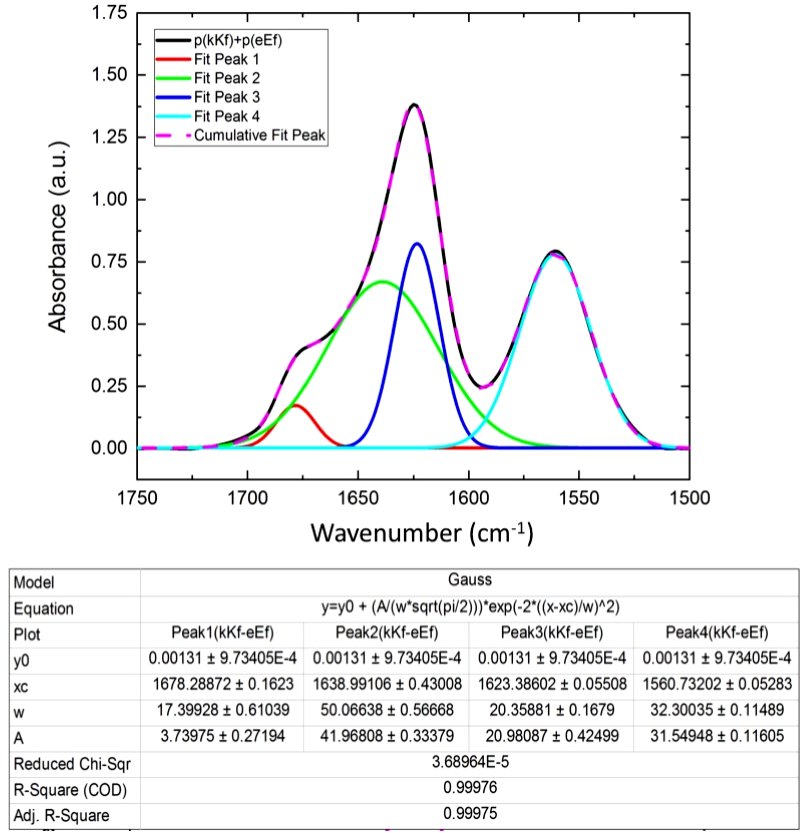

Supplement: Supplementary file 1 [file polymers-13-02074-s001.zip › Suppl. Figures/Figure S3. Deconvolution analysis of the FTIR spectra of sequence pairs (b).jpg]

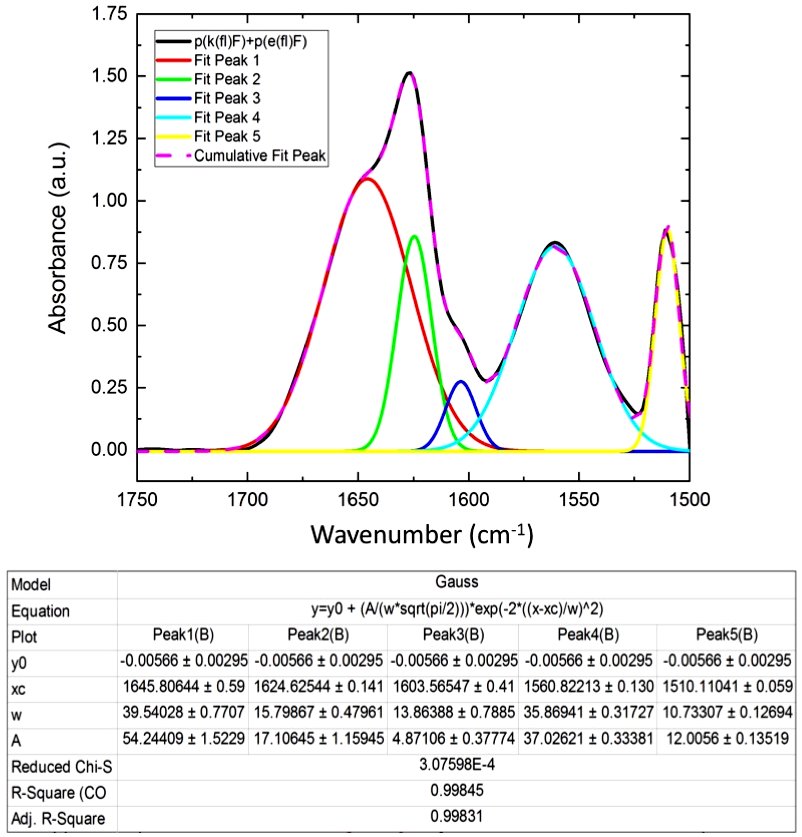

Supplement: Supplementary file 1 [file polymers-13-02074-s001.zip › Suppl. Figures/Figure S3. Deconvolution analysis of the FTIR spectra of sequence pairs (c).jpg]

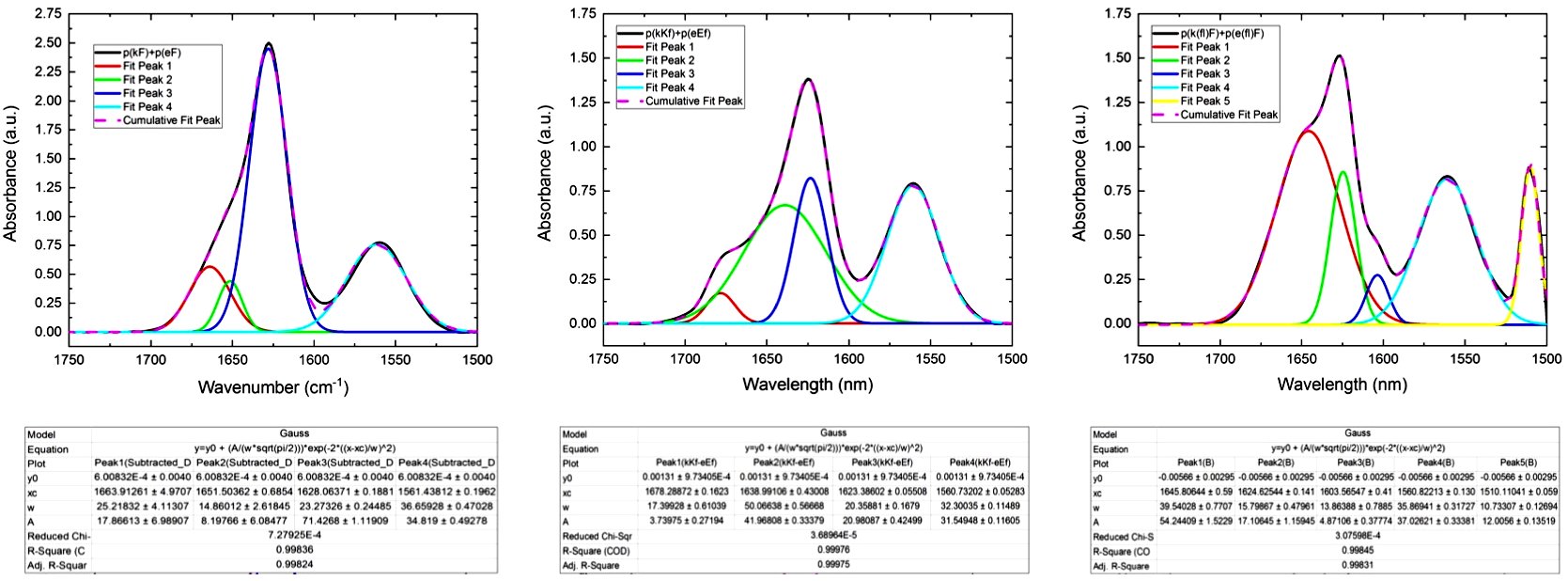

Supplement: Supplementary file 1 [file polymers-13-02074-s001.zip › Suppl. Figures/Figure S3. Deconvolution analysis of the FTIR spectra of sequence pairs.jpg]

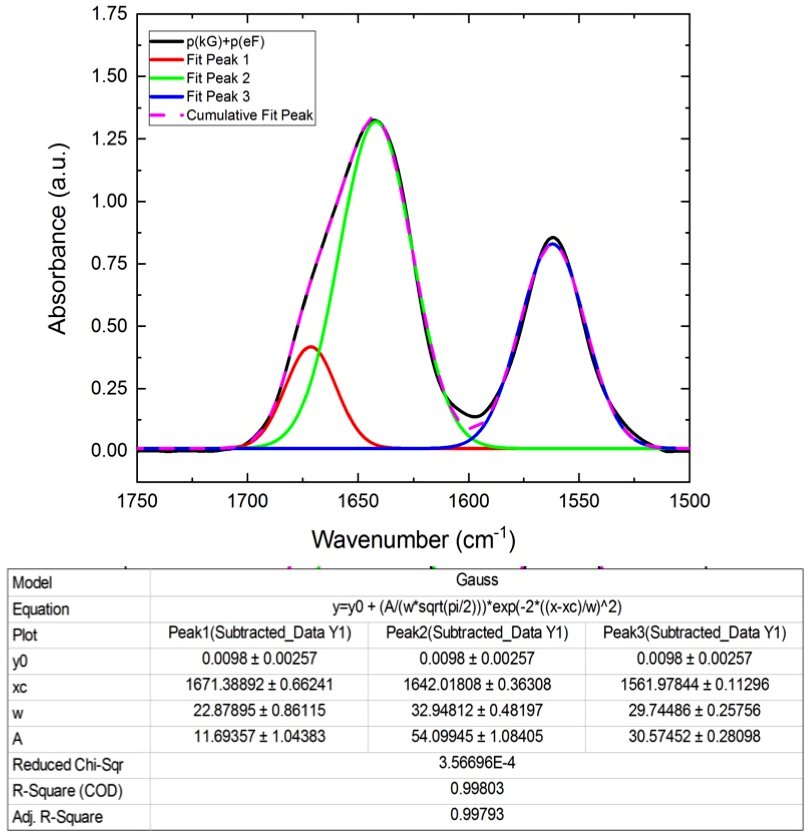

Supplement: Supplementary file 1 [file polymers-13-02074-s001.zip › Suppl. Figures/Figure S4. Deconvolution analysis of the FTIR spectra of sequence pairs (a).jpg]

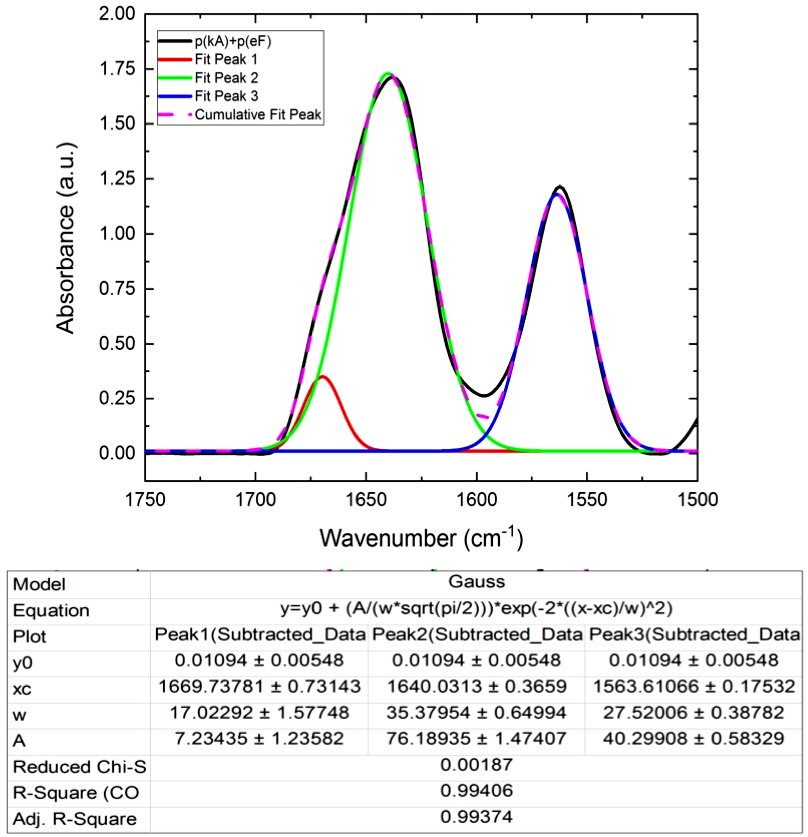

Supplement: Supplementary file 1 [file polymers-13-02074-s001.zip › Suppl. Figures/Figure S4. Deconvolution analysis of the FTIR spectra of sequence pairs (b).jpg]

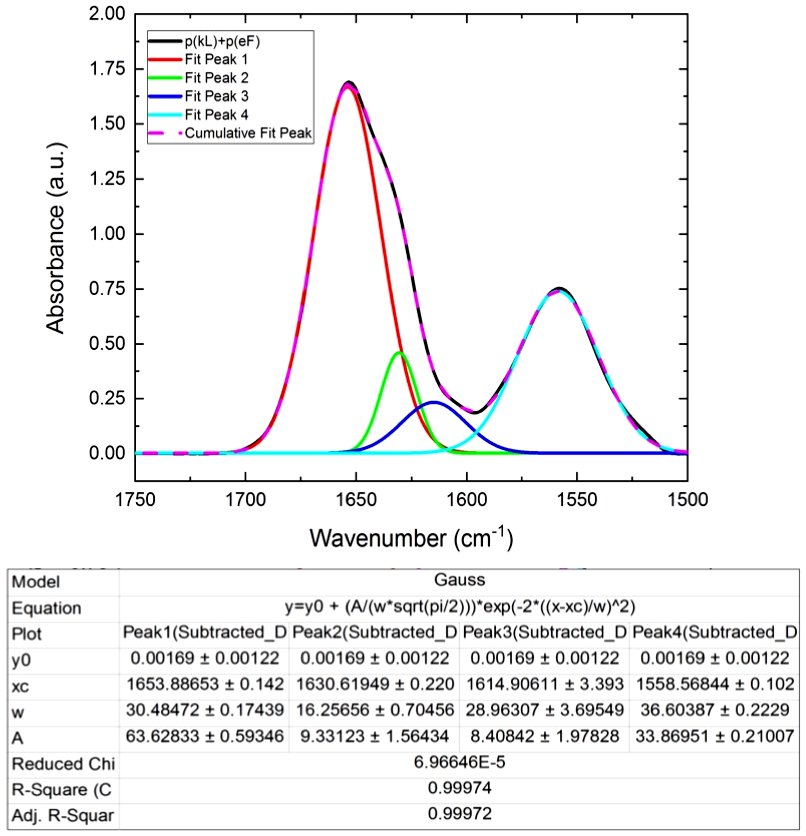

Supplement: Supplementary file 1 [file polymers-13-02074-s001.zip › Suppl. Figures/Figure S4. Deconvolution analysis of the FTIR spectra of sequence pairs (c).jpg]

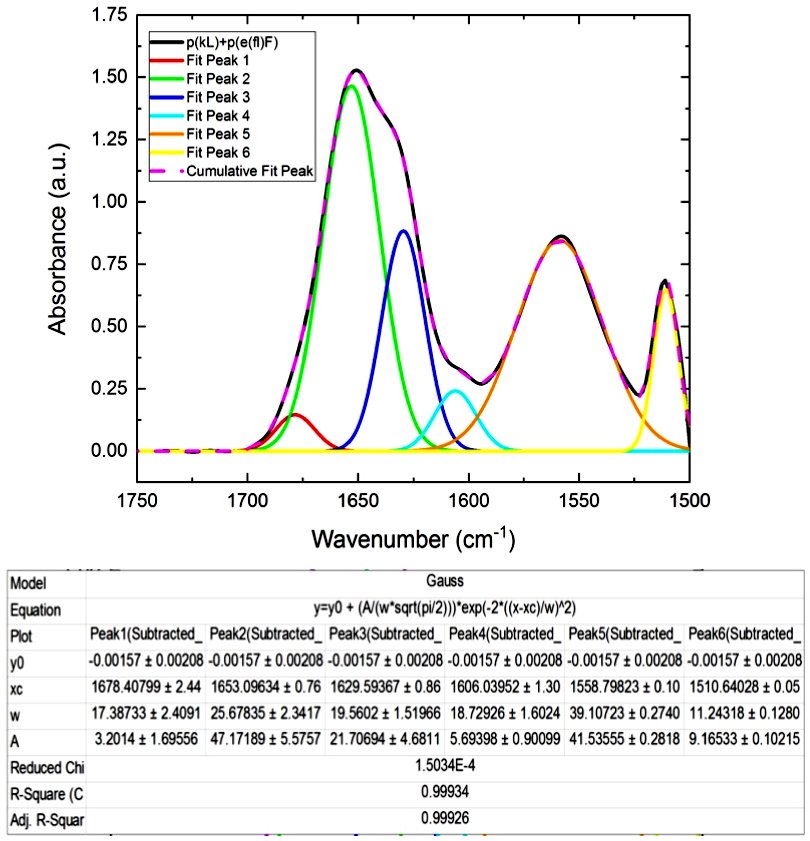

Supplement: Supplementary file 1 [file polymers-13-02074-s001.zip › Suppl. Figures/Figure S4. Deconvolution analysis of the FTIR spectra of sequence pairs (d).jpg]

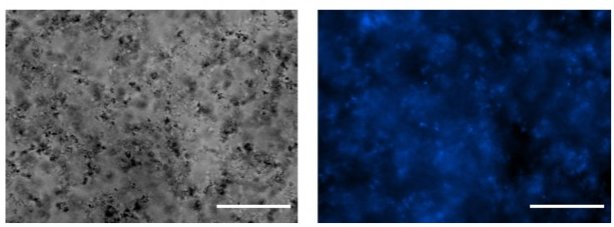

Supplement: Supplementary file 1 [file polymers-13-02074-s001.zip › Suppl. Figures/Figure S5. Optical and fluorescence imaging of p(kL)+p(e(fl)F) with thioflavin (ThT).jpg]

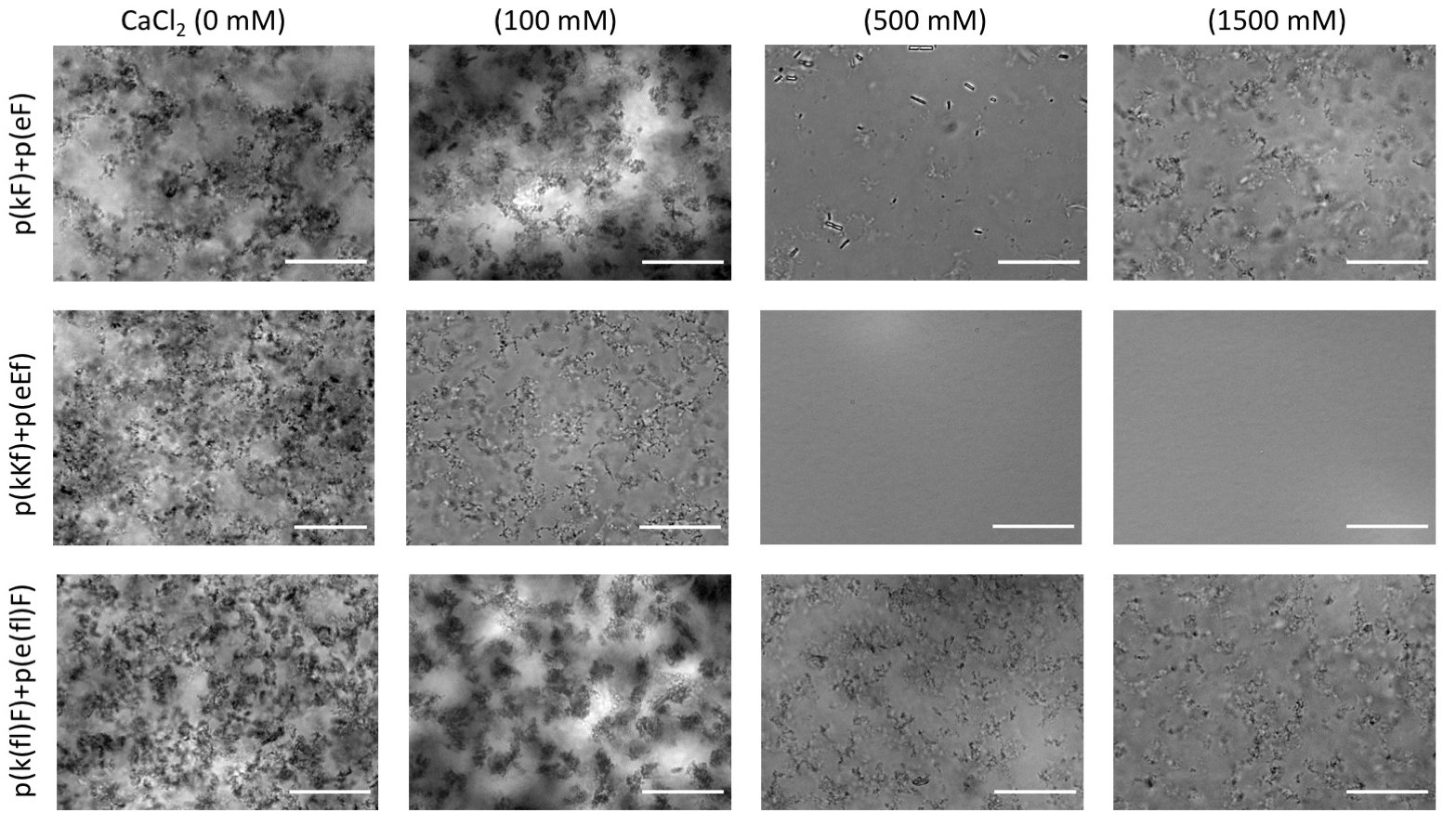

Supplement: Supplementary file 1 [file polymers-13-02074-s001.zip › Suppl. Figures/Figure S6. Optical micrographs of sequence pairs at varied salt (CaCl2) concentrations .jpg]

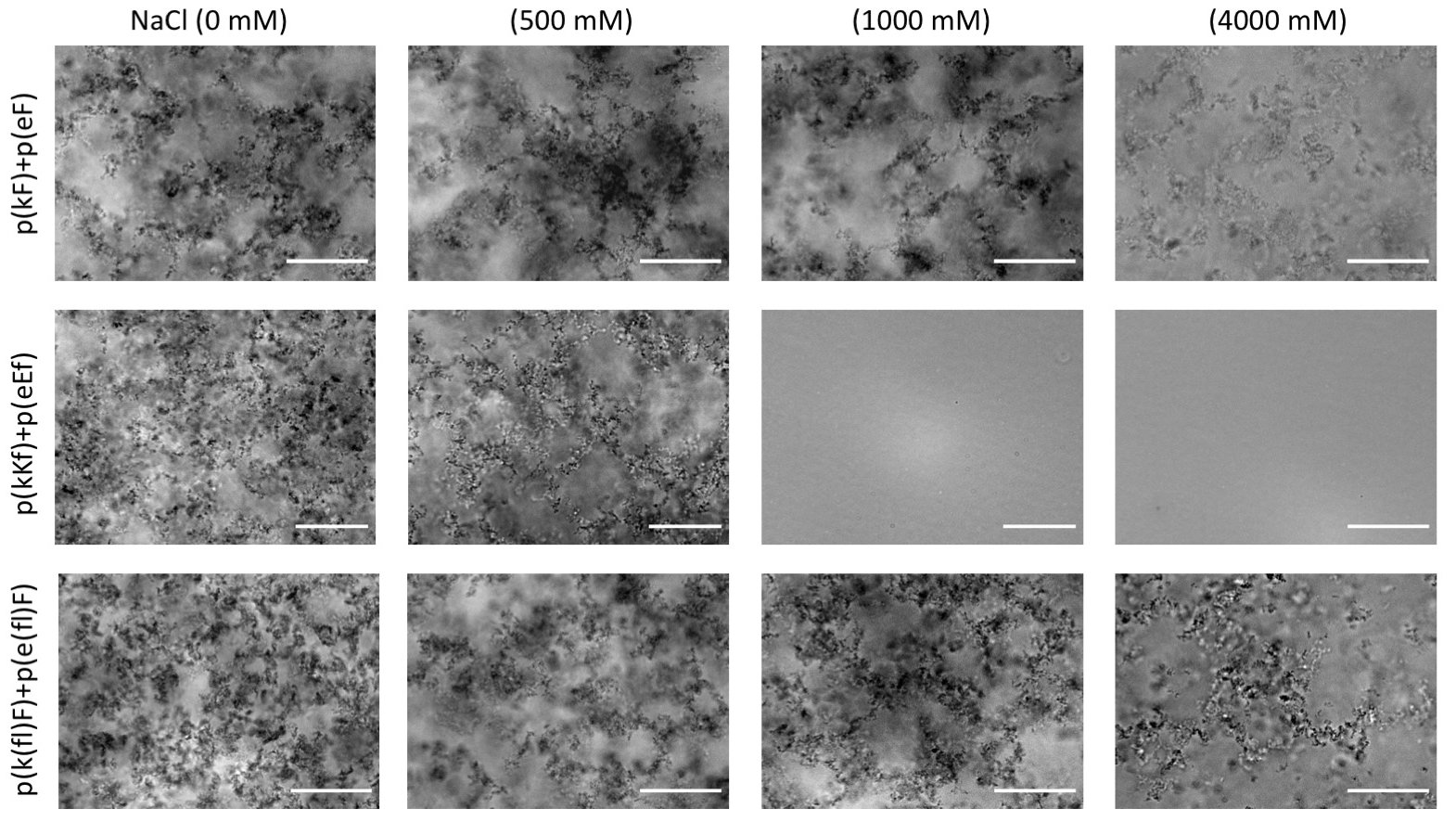

Supplement: Supplementary file 1 [file polymers-13-02074-s001.zip › Suppl. Figures/Figure S6. Optical micrographs of sequence pairs at varied salt (NaCl) concentrations .jpg]

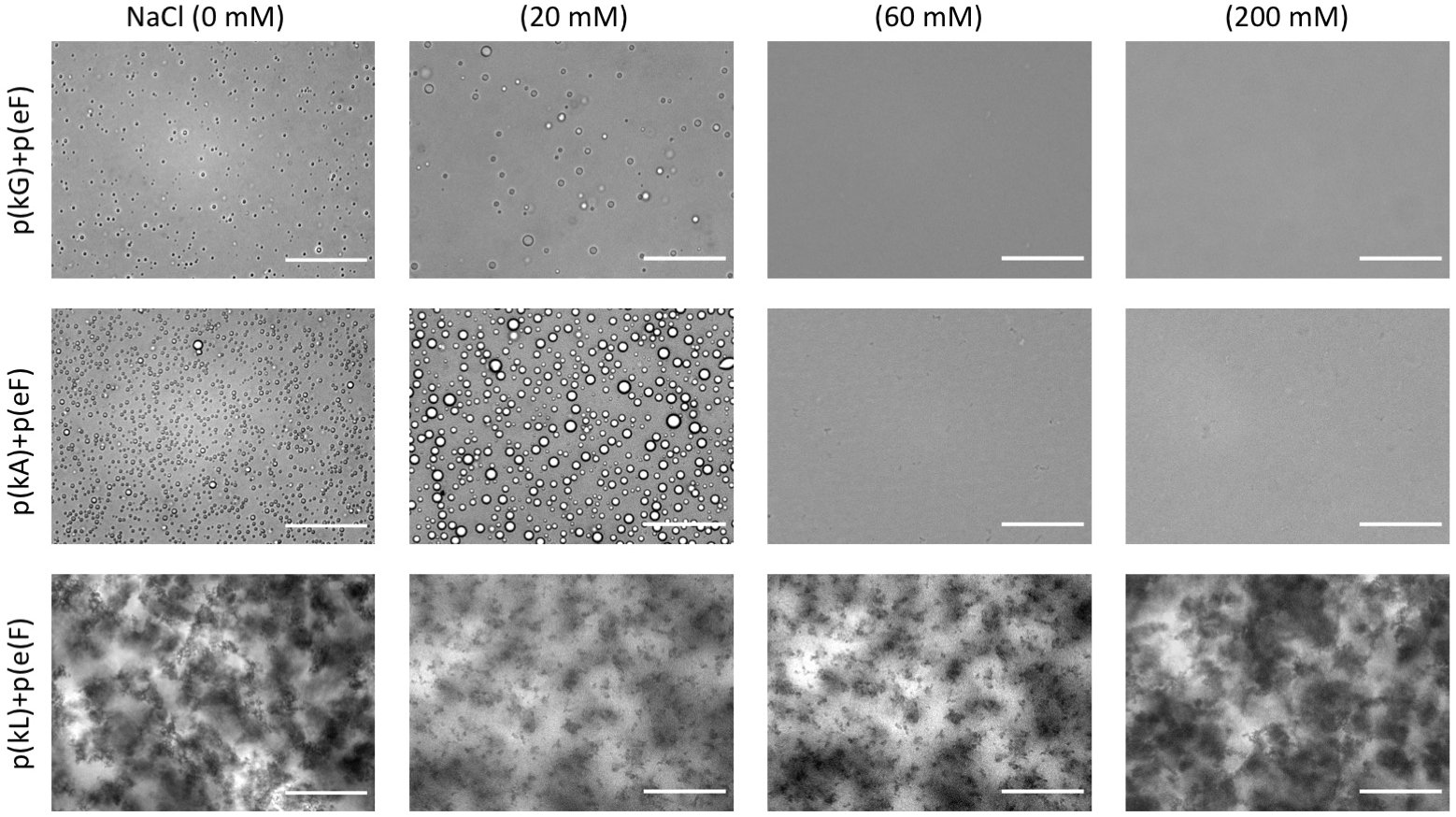

Supplement: Supplementary file 1 [file polymers-13-02074-s001.zip › Suppl. Figures/Figure S7. Optical micrographs of sequence pairs at varied salt (NaCl) concentrations .jpg]

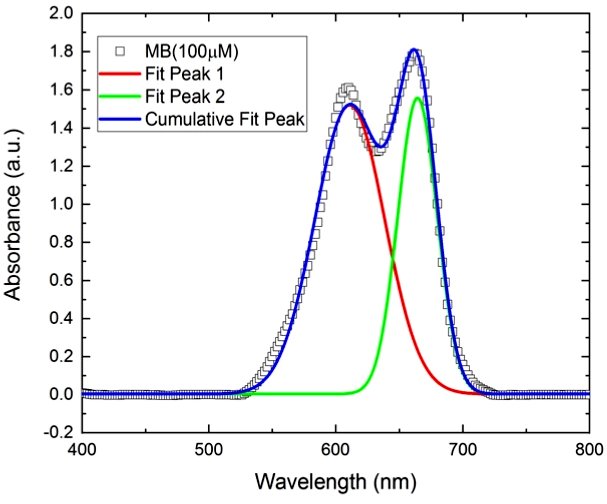

Supplement: Supplementary file 1 [file polymers-13-02074-s001.zip › Suppl. Figures/Figure S8. Deconvolution of the UV-vis spectra of methylene blue in aqueous solution and in the supernatant phase of complexes (a).jpg]

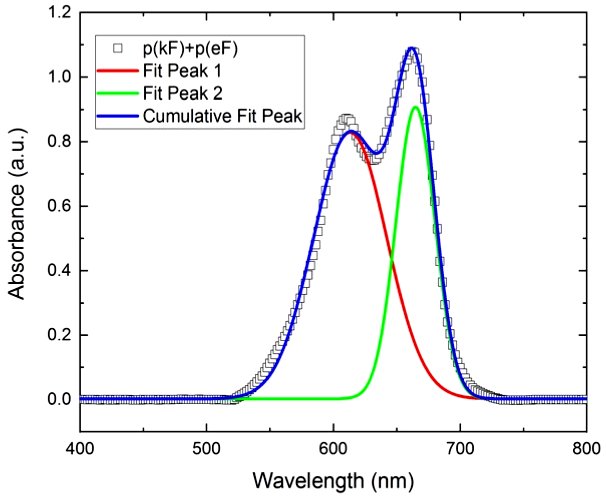

Supplement: Supplementary file 1 [file polymers-13-02074-s001.zip › Suppl. Figures/Figure S8. Deconvolution of the UV-vis spectra of methylene blue in aqueous solution and in the supernatant phase of complexes (b).jpg]

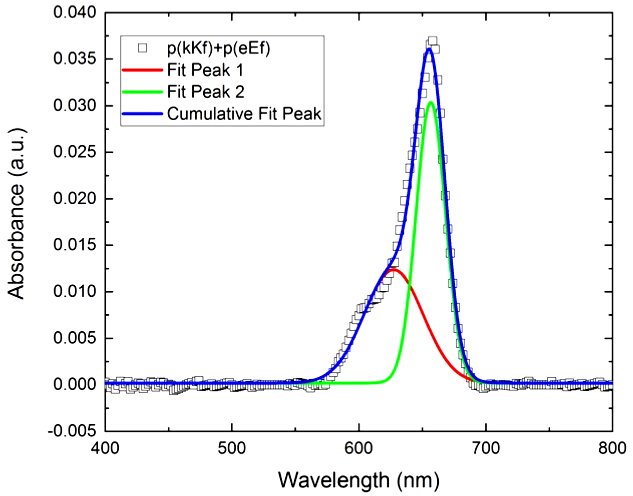

Supplement: Supplementary file 1 [file polymers-13-02074-s001.zip › Suppl. Figures/Figure S8. Deconvolution of the UV-vis spectra of methylene blue in aqueous solution and in the supernatant phase of complexes (c).jpg]

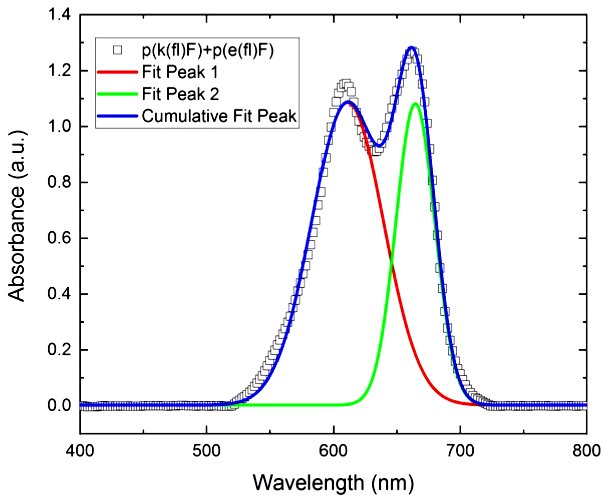

Supplement: Supplementary file 1 [file polymers-13-02074-s001.zip › Suppl. Figures/Figure S8. Deconvolution of the UV-vis spectra of methylene blue in aqueous solution and in the supernatant phase of complexes (d).jpg]

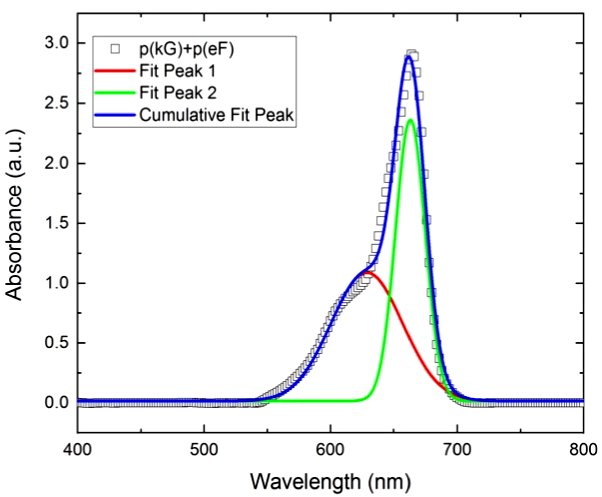

Supplement: Supplementary file 1 [file polymers-13-02074-s001.zip › Suppl. Figures/Figure S9. Deconvolution of the UV-vis spectra of methylene blue in aqueous solution and in the supernatant phase of complexes (a).jpg]

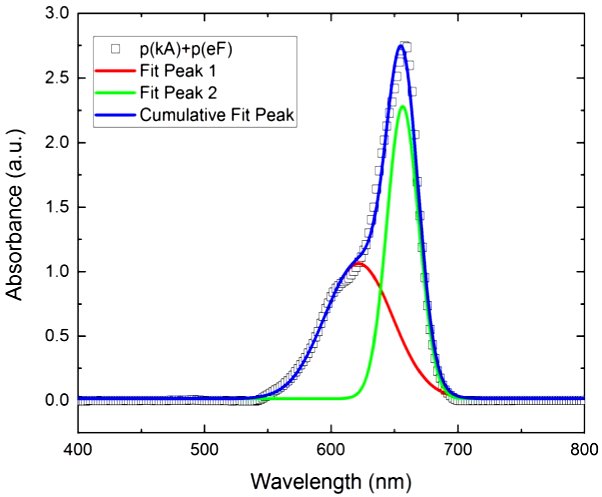

Supplement: Supplementary file 1 [file polymers-13-02074-s001.zip › Suppl. Figures/Figure S9. Deconvolution of the UV-vis spectra of methylene blue in aqueous solution and in the supernatant phase of complexes (b).jpg]

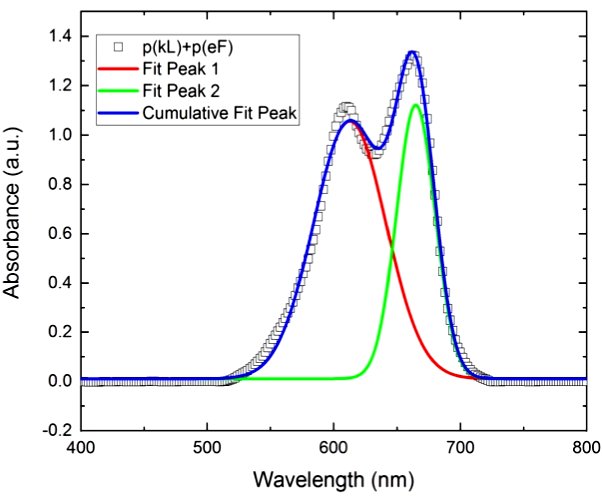

Supplement: Supplementary file 1 [file polymers-13-02074-s001.zip › Suppl. Figures/Figure S9. Deconvolution of the UV-vis spectra of methylene blue in aqueous solution and in the supernatant phase of complexes (c).jpg]

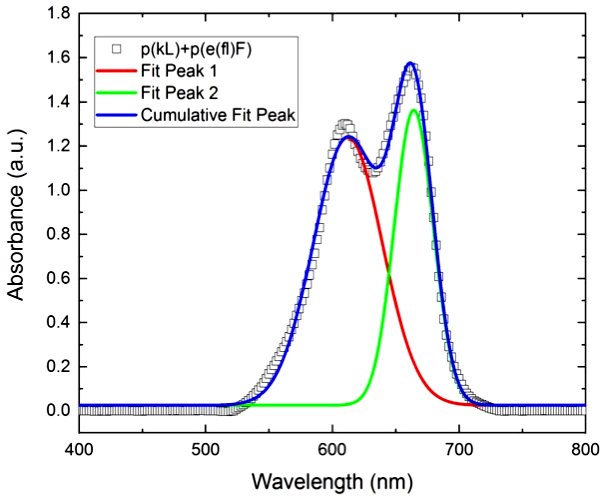

Supplement: Supplementary file 1 [file polymers-13-02074-s001.zip › Suppl. Figures/Figure S9. Deconvolution of the UV-vis spectra of methylene blue in aqueous solution and in the supernatant phase of complexes (d).jpg]
